# Functional Heterogeneity of *Fusobacterium nucleatum* Subspecies in the Immune Microenvironment of Oral Squamous Cell Carcinoma

**DOI:** 10.3390/microorganisms14092067

**Published:** 2026-09-16

**Authors:** Megumu Yano, Tomonori Kamiya, Yuki Yamamoto, Teppei Kouga, Masahiro Oishi, Miki Nishio, Akira Suzuki, Kishiko Sunami, Naoko Ohtani

**Affiliations:** 1 Department of Pathophysiology, Graduate School of Medicine, Osaka Metropolitan University, Osaka 545-8585, Japan; 2 Department of Otolaryngology and Head & Neck Surgery, Graduate School of Medicine, Osaka Metropolitan University, Osaka 545-8585, Japan; 3 Division of Molecular and Cellular Biology, Graduate School of Medicine, Kobe University, 7-5-1 Kusunoki-cho, Chuo-ku, Kobe 650-0017, Japan

**Keywords:** butyrate, *Fusobacterium nucleatum*, intratumoral bacteria, OSCC, Treg cells

## Abstract

Oral squamous cell carcinoma (OSCC) develops through multiple pathogenic and environmental factors. Increasing evidence suggests that the oral microbiota, particularly intratumoral bacteria, is also associated with OSCC progression; however, the relationship between individual bacterial subspecies and the tumor immune microenvironment remains poorly understood. We previously demonstrated that *Fusobacterium nucleatum* is enriched in OSCC tissues. In this study, we investigated the biological and immunological properties of *F. nucleatum* subspecies, with a particular focus on regulatory T-cell (Treg) induction. We reanalyzed our 16S rRNA gene sequencing dataset from 12 oral and pharyngeal squamous cell carcinoma specimens at the amplicon sequence variant (ASV) level and examined archived human OSCC tissues by immunofluorescence and fluorescence in situ hybridization (FISH). Treg differentiation and bacterial metabolite production were examined in vitro, and selected *F. nucleatum* subspecies were evaluated in a mouse tongue carcinogenesis model (*n* = 6/group). ASVs most closely matching *F. nucleatum* subsp. *animalis* were the most abundant among the detected *F. nucleatum*-associated ASVs. In three human OSCC specimens with detectable *F. nucleatum*, FOXP3-positive cells preferentially accumulated in the peritumoral stroma. In vitro, culture supernatants from *F. nucleatum* subsp. *animalis*, but not subsp. *polymorphum*, promoted Treg differentiation in two independent experiments, and butyrate production may be associated with the Treg-promoting strains. In vivo, subsp. *animalis* significantly increased Ki67-positive, nuclear YAP1-positive, and Foxp3-positive cell numbers, whereas subsp. *polymorphum* significantly increased only nuclear YAP1-positive cell numbers. Furthermore, FISH demonstrated spatial proximity between *F. nucleatum* and FOXP3-positive cells in a human OSCC case. These findings indicate functional heterogeneity among *F. nucleatum* subspecies and suggest that their effects on epithelial and immune parameters differ among subspecies. Our findings support possible subspecies-specific immunomodulatory effects of *F. nucleatum* in OSCC and highlight the importance of subspecies- and strain-level analyses for understanding the biological significance of the intratumoral microbiota.

## 1. Introduction

Oral squamous cell carcinoma (OSCC) is the most common malignancy of the oral cavity and is strongly associated with well-established risk factors, including tobacco use, alcohol consumption, poor oral hygiene, chronic mechanical irritation, and chronic inflammation. However, these classical risk factors alone cannot fully explain the development and progression of OSCC, and increasing evidence suggests that the oral microbiota and microbiota-associated alterations in host immune responses contribute to oral carcinogenesis. The human oral cavity comprises diverse ecological niches, including the tooth surface, gingival sulcus, tongue, and buccal mucosa, each of which harbors a distinct microbial community. While the oral microbiota is essential for maintaining oral homeostasis, disruption of the microbial community (oral dysbiosis) has been implicated in the pathogenesis of various oral diseases, including periodontitis and OSCC [1].

In recent years, bacteria have been identified within tumor tissues across multiple cancer types, revealing the intratumoral microbiota as an important component of the tumor microenvironment. In colorectal cancer, Fusobacterium species are frequently enriched within tumor tissues and have been implicated in tumor progression [2,3]. Furthermore, pan-cancer analyses have demonstrated that intratumoral microbiota exhibit cancer type-specific compositions and that bacteria are present not only within tumor cells but also within infiltrating immune cells [4]. Accumulating experimental evidence further indicates that intratumoral bacteria can influence therapeutic resistance, metastasis, inflammatory responses, antitumor immunity, and patient prognosis [5,6,7,8]. Together, these findings suggest that intratumoral bacteria may influence tumor biology through interactions with the tumor microenvironment.

*Fusobacterium nucleatum* is an obligately anaerobic, Gram-negative bacillus that resides in the oral cavity and is involved not only in periodontitis and oral biofilm formation but also has been reported to be associated with carcinogenesis and tumor progression, particularly in colorectal cancer. In colorectal cancer and related experimental models, *F. nucleatum* activates the E-cadherin/β-catenin signaling pathway through FadA [9] and accumulates in tumor tissues by binding to glycans on the surface of tumor cells through Fap2 [10]. Furthermore, Fap2 can suppress antitumor immune responses mediated by NK cells and T cells through its interaction with TIGIT [11]. Importantly, *F. nucleatum* is not a homogeneous bacterial species but consists of multiple subspecies and genetically distinct strain populations. *F. nucleatum* subsp. *animalis* has been shown to induce inflammatory responses and macrophage activation and to promote colorectal tumorigenesis [12]. More recently, specific lineages belonging to *F. nucleatum* subsp. *animalis* have been reported to be enriched in colorectal cancer tissues, suggesting that the tumor tropism and immunomodulatory properties of *F. nucleatum* may be determined at the subspecies or strain level rather than at the species level [13]. In OSCC, associations between the intratumoral microbiota, including *F. nucleatum*, and the tumor immune microenvironment have also begun to be reported. The abundance of *F. nucleatum* in tumor tissues has been reported to be associated with clinicopathological factors, prognosis, and the expression of immune-related genes [14]. In addition, it has been suggested that the intratumoral microbiota changes during the progression of OSCC and may be associated with immune responses [15]. However, it remains unclear whether *F. nucleatum* plays a similar role in OSCC as it does in other cancer types, and its effects may vary depending on the cancer type, intratumoral localization, the coexisting microbiota, and subspecies- or strain-level differences.

Furthermore, cancer progression is largely determined not only by tumor cells but also by the tumor microenvironment (TME), which is composed of immune cells, fibroblasts, vascular endothelial cells, and other cellular components. Oral squamous cell carcinoma (OSCC) arises in the context of chronic inflammation and persistent exposure to oral bacteria, and in addition to the modulation of host immune responses by oral bacteria and their metabolites, recent studies have suggested that the intratumoral microbiota may also influence the formation of the immune microenvironment and antitumor immune responses. Under such conditions, immune cells and stromal cells interact in a complex manner, leading to the formation of an immunosuppressive tumor microenvironment. This microenvironment is characterized by the accumulation of immunosuppressive cells, including regulatory T cells (Tregs), tumor-associated macrophages (TAMs), and myeloid-derived suppressor cells (MDSCs), which are thought to contribute to the suppression of antitumor immune responses [16,17]. Among these, Tregs are a representative immunoregulatory cell population characterized by the expression of the transcription factor Foxp3 and can suppress antitumor immune responses. The accumulation of Tregs within the tumor contributes to immune evasion and has been reported to be associated with poor prognosis in many solid cancers [18]. Although Treg infiltration has also been reported to be associated with prognosis and clinicopathological factors in OSCC, its role has been suggested to differ depending on its intratumoral localization and functional state, and its biological significance has not yet been fully elucidated [19]. The biological and clinical significance of FOXP3-positive cells in OSCC may also vary according to tumor stage, anatomical site, HPV status, and the surrounding immune-cell composition, and FOXP3 expression alone does not necessarily establish their functional immunosuppressive activity.

Bacteria can be involved in Treg induction through bacterial metabolites, bacterial components, alterations in the function of antigen-presenting cells, and changes in the cytokine milieu. Indeed, bacterial metabolites, including short-chain fatty acids such as butyrate and propionate, promote FOXP3 expression and peripheral Treg differentiation [20,21,22], and specific groups of commensal bacteria have also been shown to induce Tregs at mucosal sites [23,24]. Therefore, intratumoral bacteria present in the OSCC microenvironment may contribute to the establishment of a Treg-rich immune microenvironment. However, it remains unclear how *F. nucleatum* and differences at the subspecies and strain levels influence Treg accumulation and the tumor immune microenvironment in OSCC.

In the present study, we focused on the relationship between intratumoral bacteria and the immune microenvironment, particularly Tregs, in OSCC. Based on our previous study suggesting a role for *F. nucleatum* subsp. *polymorphum* in OSCC progression, we compared its biological and immunological properties with those of *F. nucleatum* subsp. *animalis*, which was selected based on the ASV-level reanalysis performed in the present study. We further examined Treg-promoting activity and bacterial metabolite production in vitro and evaluated the effects of selected *F. nucleatum* subspecies on epithelial and immune parameters in a mouse tongue carcinogenesis model.

## 2. Materials and Methods

### 2.1. Ethics

All human sample analyses in the present study utilized data and specimens from our previously published cohort [25]. The original study was approved by the Ethics Committee of Osaka Metropolitan University (approval numbers: 2020-079 and 2021-282) in accordance with the Declaration of Helsinki, and all patients and participants provided written informed consent. All mouse experiments were approved by the Animal Care and Use Committee of Osaka Metropolitan University (approval number: 22012).

### 2.2. Patients

The patient cohort used in the present study has been described previously [25]. Briefly, the study was conducted at the Department of Otolaryngology and Head and Neck Surgery, Osaka Metropolitan University Hospital, Osaka, Japan, between April 2020 and August 2023. Sixty-five patients with oral and pharyngeal cancer (diagnosed as squamous cell carcinoma, SCC) and ninety-three control patients without oral cancer were included. The exclusion criteria were age < 20 years, a history of radiotherapy, antibiotic treatment, p16^INK4a^ positivity, indication of human papillomavirus (HPV) infection in the tumor area, tumors other than histologically squamous cell carcinoma (SCC), and head and neck cancers developed in the larynx. Among the 65 patients with oral and pharyngeal cancer, fresh tumor biopsy specimens were obtained before treatment. After securing the tissue required for pathological diagnosis, sufficient residual biopsy tissue for bacterial 16S rRNA gene analysis was available from 12 patients. These 12 cases were therefore included in the tumor-associated microbiota analysis and were not selected based on specific clinical or microbiological characteristics. Because inclusion depended on the availability of sufficient residual biopsy tissue, these 12 cases did not constitute a strictly consecutive series. The present ASV-level reanalysis was restricted to these 12 tumor specimens. The clinical characteristics of these 12 patients are summarized in Table 1.

**Table 1:** Clinical characteristics of the 12 patients included in the tumor-associated microbiota analysis. Clinical and pathological characteristics of the 12 patients with oral and pharyngeal squamous cell carcinoma included in this study. Patient information corresponding to the samples analyzed in Figure 1 is summarized.

**Patient’s_ID**	**Age**	**Sex**	**Diagnosis**	**p16**	**Recurrence**	**Head and Neck**	**Esophagus**	**Chemotherapy**			
T001	75	F	tongue	−	−	−	−	+			
T002	77	M	tongue	−	−	−	−	+			
T010	79	M	tongue	−	−	−	−	−			
T012	77	F	oral	−	−	−	−	−			
T023	84	F	oral	−	−	−	−	+			
T032	55	M	oropharynx	−	−	−	−	−			
T091	33	M	tongue	−	−	−	−	+			
T092	45	F	tongue	−	−	−	−	−			
T103	57	M	oral	−	−	−	−	−			
T110	89	F	tongue	−	−	−	+	−			
T111	61	F	tongue	−	−	−	−	−			
T116	80	F	tongue	−	−	−	−	−			
**Patient’s_ID**	**Denture**	**Radiation**	**Antibiotic**	**PPI**	**Intestinal Regulator**	**Drinking**	**Smoking**	**T**	**N**	**M**	**Stage**
T001	+	−	−	−	−	−	+	3	0	0	III
T002	−	−	−	+	−	−	+	3	0	0	III
T010	+	−	−	−	−	−	+	2	0	0	II
T012	−	−	−	+	−	−	−	2	1	0	III
T023	−	−	−	+	−	−	−	2	0	0	II
T032	−	−	−	−	−	−	+	2	0	0	II
T091	−	−	−	+	+	−	−	2	0	0	II
T092	−	−	−	−	−	−	−	3	0	0	III
T103	−	−	−	+	−	−	+	4a	2b	0	IVa
T110	−	−	−	−	−	−	−	2	0	0	II
T111	−	−	−	−	−	+	+	2	0	0	II
T116	−	−	−	+	−	−	−	2	0	0	II

+: positive; −: negative.

### 2.3. Human Tissue Samples

In the present study, formalin-fixed, paraffin-embedded (FFPE) tissue blocks from patients with oral and pharyngeal cancer, originally prepared and archived by the Department of Otolaryngology and Head and Neck Surgery, Osaka Metropolitan University Hospital, for routine pathological diagnosis, were utilized for immunofluorescence and Fluorescence In Situ Hybridization (FISH) analyses. Details of sample collection and storage for the previously published microbiota dataset have been described elsewhere [25].

### 2.4. DNA Extraction and 16S rRNA Gene Sequencing

As described previously [25], the V1–V2 hypervariable region of the bacterial 16S rRNA gene was amplified using the primers 27Fmod (5′-AGRGTTTGATCMTGGCTCAG-3′) and 338R (5′-TGCTGCCTCCCGTAGGAGT-3′), and sequencing was performed using 250-bp paired-end reads on a MiSeq system (Illumina, San Diego, CA, USA). Oral microbiota profiling data from our previously published dataset [25] were reanalyzed in the present study using the QIIME2 pipeline (version 2021.11) with DADA2 denoising and the Silva 138 99% OTU classifier. Tumor-associated microbiota sequencing data from 12 patients with oral and pharyngeal cancer were subjected to amplicon sequence variant (ASV)-level reanalysis using NCBI BLASTn searches (https://blast.ncbi.nlm.nih.gov, accessed 23 January 2024). ASVs detected at a relative abundance of ≥0.1% in each tumor tissue sample were extracted, and those meeting the criteria of ≥97% identity and ≥95% coverage were further analyzed based on their BLASTn results. For Fusobacterium-associated ASVs, the ASV length and the top five BLASTn matches, including query coverage, percent identity, E-value, and reference accession number, were examined, and the taxonomic designation used in the present study was based on the top BLASTn hit. Detailed BLASTn results are provided in Supplementary Figure S2. Because short 16S rRNA amplicon sequences may not reliably distinguish closely related *F. nucleatum* subspecies, subspecies-level assignments were interpreted cautiously and are described as ASVs “most closely matching” the corresponding subspecies where appropriate. For ASVs classified into the same species, relative abundances were summed to calculate species-level abundance within tumor tissues.

### 2.5. Fluorescence in Situ Hybridization (FISH)

FISH was carried out on formalin-fixed, paraffin-embedded (FFPE) oral cancer tissue samples. Sections were hybridized with the 5′-Alexa Fluor 488-labeled universal bacterial probe EUB338 (5′-GCTGCCTCCCGTAGGAGT-3′) [26] and the 5′-Texas Red-labeled Fusobacterium nucleatum-specific probe FUSO664 (5′-CTTGTAGTTCCGCYTACCTC-3′; pB-1346, http://probebase.csb.univie.ac.at(accessed on 11 October 2023)) [25]. Slides were deparaffinized and treated with wash buffer (0.9 M NaCl, 20 mM Tris-HCl [pH 7.5], and 0.1% SDS) for 60 min at 42 °C. Slides were then hybridized overnight with the specified FISH probes at a concentration of 2 μM in hybridization buffer (0.9 M NaCl, 20 mM Tris-HCl [pH 7.5], 0.01% SDS, and 20% formamide) at 42 °C. Post-hybridization, slides were rinsed for 60 min at 42 °C in wash buffer. Tissue sections were counterstained with DAPI and mounted. Images were captured using an IX71 fluorescence microscope (Olympus, Tokyo, Japan).

### 2.6. Bacterial Strains and Culture Conditions

*Fusobacterium nucleatum* reference strains were obtained from the Japan Collection of Microorganisms (JCM), Microbe Division, RIKEN BioResource Research Center (Tsukuba, Japan; https://jcm.brc.riken.jp/en/ (accessed on 4 April 2024)). Strains were cultured on modified Gifu Anaerobic Medium (GAM) agar plates (Nissui, Tokyo, Japan) and maintained in modified GAM broth under strictly anaerobic conditions (85% N_2_, 5% H_2_, 10% CO_2_) at 37 °C for 24 h. To prepare conditioned culture supernatants, bacterial cultures grown to confluence were centrifuged at 3000× *g* for 15 min at room temperature. The supernatants were carefully collected, aliquoted, and stored at −80 °C until use. For experimental inoculation in mouse studies, the bacterial pellet was washed twice with PBS and resuspended in PBS containing 2% carboxymethyl cellulose at approximately 1 × 10^9^ CFU/50 μL.

### 2.7. Naive CD4^+^ T Cell Isolation and Regulatory T Cell Induction

Naive CD4^+^ T cells were isolated from the spleens and lymph nodes of C57BL/6N mice. Briefly, harvested spleens and lymph nodes were dissociated, and red blood cells were lysed using RBC Lysis Buffer (BioLegend, San Diego, CA, USA). The cell suspension was washed with 2% FBS/PBS and incubated with anti-mouse CD16/32 (clone 2.4G2, Selleck, Houston, TX, USA) to block Fc receptors. Cells were then labeled with a biotinylated antibody cocktail targeting lineage markers—anti-CD11b (clone M1/70), anti-CD11c (clone N418), anti-CD44 (clone IM7), anti-CD49b (clone DX5), anti-CD19 (clone 6D5), and anti-TER119 (clone TER-119) (all BioLegend, San Diego, CA, USA)—followed by incubation with MojoSort Streptavidin Nanobeads (BioLegend). Naive CD4^+^ T cells were negatively selected by MojoSort magnetic separation and subsequently sorted by flow cytometry using the gating strategy: PI^−^CD3ε^+^ (clone 145-2C11) CD4^+^ (clone GK1.5) CD25^−^ (clone PC61) CD44^−^ (clone IM7) CD8α^−^ (clone 53-6.7) (all BioLegend).

For Treg induction, sorted naive CD4^+^ T cells were cultured on plates coated with anti-CD3ε (clone 145-2C11, 1 μg/mL, BioLegend) and anti-CD28 (clone 37.51, 1 μg/mL, BioLegend) in the presence of recombinant mouse IL-2 (10 ng/mL, BioLegend) and recombinant mouse TGF-β (0.2 ng/mL, BioLegend) for 3 days at 37 °C under 5% CO_2_. Where indicated, bacterial culture supernatant was added at a 1:40 dilution at the initiation of culture. A medium-only condition without bacterial culture supernatant was included as a control. After 3 days, cells were harvested and surface-stained with anti-CD3ε (clone 145-2C11) and anti-CD4 (clone GK1.5) (all BioLegend), with Zombie NIR (BioLegend) positive exclusion for live/dead discrimination. Intracellular Foxp3 staining was performed using the Foxp3/Transcription Factor Staining Buffer Set (Thermo Fisher Scientific, Waltham, MA, USA) according to the manufacturer’s instructions, with anti-Foxp3 (clone FJK-16s, Thermo Fisher Scientific). Treg induction efficiency was assessed by calculating the percentage of Foxp3^+^ cells within the Zombie NIR^−^CD3ε^+^CD4^+^ gate. The flow cytometric gating strategy used for the Treg differentiation assay is shown in Supplementary Figure S3A.

Treg differentiation assays using bacterial culture supernatants were independently performed twice. In each independent experiment, T cells were isolated from approximately four donor mice, and bacterial culture supernatants were prepared from independent bacterial cultures. Each experimental condition was analyzed in triplicate wells, which were considered technical replicates. To assess the effect of butyrate on Treg differentiation, sodium butyrate was added at 0, 0.5, 2, or 10 mM at the initiation of culture under the same conditions described above. These experiments were independently performed twice.

### 2.8. Short-Chain Fatty Acid Quantification by LC-MS/MS

The concentrations of short-chain fatty acids (SCFAs), acetic acid, propionic acid, butyric acid, valeric acid, isovaleric acid, lactic acid, and pyruvic acid in bacterial culture supernatants were quantified by liquid chromatography–tandem mass spectrometry (LC-MS/MS) using a Shimadzu LCMS-8060 system (Shimadzu Corporation, Kyoto, Japan) with the Short-Chain Fatty Acid Method Package (Shimadzu Corporation) according to the manufacturer’s instructions. Culture supernatants were prepared as described in Section 2.6. Samples were derivatized following the standard protocol provided in the method package, and SCFA concentrations were calculated from calibration curves generated with authentic standards. The LC-MS/MS analysis was performed once.

### 2.9. Conditional Mob1a/b DKO (YAP-Activated) Mice as a Tongue Cancer Model

The generation and tamoxifen-induced activation of Rosa26-CreERT; Mob1a^flox/flox^; Mob1b^−/−^ mice have been described previously [27]. Briefly, tamoxifen (10 mg/mL in 100% ethanol, 15 μL) was applied to the tongue daily for 5 days beginning at 4 weeks of age under anesthesia with pentobarbital and isoflurane. From 5 weeks of age, mice received drinking water supplemented with sulfamethoxazole (1.6 mg/mL) and trimethoprim (0.32 mg/mL) for 5 days. From 6 weeks of age, mice were administered either PBS (vehicle), *F. nucleatum* subsp. *animalis*, or *F. nucleatum* subsp. *polymorphum* to the tongue every 3 days for a total of eight administrations. The JCM-derived *F. nucleatum* strains prepared as described in Section 2.6 were applied to the tongues at approximately 1 × 10^9^ CFU in 50 μL of PBS containing 2% carboxymethyl cellulose per administration. Tongue tissues were collected on the day following the final administration and subjected to histological and immunohistochemical analyses as described in Section 2.10. Six mice per group were used for the quantitative analyses presented in this study, and each individual mouse was considered an independent biological replicate. An a priori sample-size calculation was not performed because the number of mice with the required compound genotype was limited by breeding yield. No animals were excluded from the analysis after initiation of the experiment. Formal randomization and blinded assessment were not performed. Eligibility was based on the required compound genotype and experimental age, with no additional eligibility criteria applied. Because formal randomization was not performed, allocation concealment was not applicable.

### 2.10. Immunostaining

For human samples, immunofluorescence staining was performed on FFPE oral cancer tissue sections. Sections were deparaffinized, rehydrated, and subjected to heat-induced antigen retrieval for 20 min using Retrieval Solution (S1699, Dako, Santa Clara, CA, USA). After blocking with a blocking reagent (HK085-5K, BioGenex, San Ramon, CA, USA) for 15 min at room temperature, sections were co-incubated overnight at 4 °C with anti-CK17 (polyclonal, rabbit, Proteintech, Rosemont, IL, USA) and anti-FOXP3 (clone 237A/E7, mouse, Thermo Fisher Scientific Inc., Waltham, MA, USA) primary antibodies. To further characterize FOXP3-positive cells, additional sections were co-stained with anti-CD4 (clone EPR6855, rabbit, Abcam, Cambridge, UK) and anti-FOXP3 (clone 237A/E7). Sections were then incubated with Alexa Fluor 488-conjugated donkey anti-mouse IgG (A-21202, Invitrogen, Carlsbad, CA, USA) and Alexa Fluor 594-conjugated donkey anti-rabbit IgG (A-21207, Invitrogen) secondary antibodies for 1 h at room temperature, and counterstained with DAPI (19178-91, Nacalai, Kyoto, Japan). Sections were mounted with ProLong™ Gold Antifade Mountant (P36930, Invitrogen).

For quantitative analysis of FOXP3-positive cells, three OSCC specimens in which *F. nucleatum* was detected by 16S rRNA analysis were analyzed. In each specimen, five fields were evaluated for each of three histological compartments: adjacent non-neoplastic epithelium, intratumoral epithelial regions, and peritumoral stroma. Each patient was considered an independent biological sample, whereas the five fields evaluated within each histological compartment were treated as nested measurements and were not considered independent biological replicates. Intratumoral epithelial regions were defined based on CK17-positive tumor epithelial areas, whereas peritumoral stroma was defined as CK17-negative stromal areas immediately adjacent to CK17-positive tumor nests. Adjacent non-neoplastic epithelium was identified morphologically in areas without neoplastic changes. FOXP3-positive cells were counted in each field and expressed as the number of FOXP3-positive cells per field.

For mouse samples, tongue tissues were fixed in 4% paraformaldehyde, embedded in paraffin, and sectioned at 5 μm. Sections were deparaffinized, rehydrated, and subjected to heat-induced antigen retrieval for 20 min using Retrieval Solution (S1699, Dako). After blocking with a blocking reagent (HK085-5K, BioGenex) for 15 min at room temperature, sections were individually incubated overnight at 4 °C with one of the following primary antibodies: anti-Ki67 (RM-9106-S0, Thermo Fisher Scientific Inc.), anti-YAP1 (#4912S, Cell Signaling Technology Inc., Beverly, MA, USA), or anti-Foxp3 (clone FJK-16s, Thermo Fisher Scientific Inc.). Sections were then incubated with a peroxidase-conjugated secondary antibody (MK202, Takara, Kusatsu, Japan) and developed with nickel-enhanced 3,3′-diaminobenzidine (DAB, MK210, Takara), followed by counterstaining with Mayer’s Hematoxylin. Sections were mounted with Entellan™ new (1.07961.0100, Sigma-Aldrich, St. Louis, MO, USA).

All images were captured using a BZ-X800 fluorescence microscope (Keyence, Osaka, Japan) and analyzed with BZ-X software version 1.1.1.8 (Keyence). For Ki67- and YAP1-positivity assessments, the entire tongue epithelial area of each mouse was measured using BZ-X software, and the proportion of the positive region within the epithelial area was determined. For Foxp3-positivity assessments, positive cells were counted within the tongue epithelial area and the surrounding peribasal membrane region. Six mice per group from the main experiment were included in the quantitative Ki67, YAP1, and Foxp3 analyses, with each individual mouse considered an independent biological replicate.

### 2.11. Biofilm Formation Assay

Biofilm formation was evaluated using a crystal violet staining assay in sterile flat-bottom 96-well polystyrene microplates (Corning Costar 3596, Corning Inc., Corning, NY, USA). A single bacterial colony was inoculated into modified Gifu Anaerobic Medium (mGAM) broth (Nissui Pharmaceutical, Tokyo, Japan) and cultured overnight under anaerobic conditions. The culture was adjusted to an optical density at 600 nm (OD600) of 0.1–0.2.

For the initial biofilm formation, 150 μL of the bacterial suspension was dispensed into each well and incubated statically at 37 °C for 24 h under anaerobic conditions (85% N2, 10% CO_2_, and 5% H_2_) using an anaerobic chamber (Concept 400; Baker Ruskinn, Bridgend, UK). After incubation, the culture supernatant was carefully aspirated without disturbing the attached biofilm, and each well was gently washed three times with 200 μL of sterile physiological saline.

Subsequently, one of the following inoculation conditions was applied: (i) fresh medium containing a single bacterial suspension adjusted to OD600 = 0.1–0.2 (150 μL/well) or (ii) fresh medium containing an equal-volume mixture of two bacterial suspensions (1:1, *v*/*v*), each adjusted to OD600 = 0.1–0.2, with a final volume of 150 μL per well. Plates were further incubated statically for 72 h under the same anaerobic conditions.

Following incubation, the culture medium was removed, and each well was gently washed three times with 200 μL of sterile physiological saline. The attached biofilm was stained with 150 μL of 0.1% (*w*/*v*) crystal violet solution (prepared from crystal violet powder; FUJIFILM Wako Pure Chemical Corporation, Osaka, Japan) for 1 h at room temperature. Excess stain was removed by washing the wells three times with sterile physiological saline, after which the bound crystal violet was solubilized with 150 μL of 100% ethanol for 30 min. The ethanol extracts were diluted fivefold with 100% ethanol prior to absorbance measurement. Biofilm biomass was quantified by measuring the absorbance at 595 nm (OD595) using an iMark Microplate Reader (Bio-Rad Laboratories, Hercules, CA, USA). The measured OD595 values were multiplied by five to obtain the final OD595 values. The biofilm formation assay was independently performed four times. In each independent experiment, each bacterial condition was analyzed in triplicate wells as technical replicates, and the mean of the triplicate wells was used as one independent measurement.

### 2.12. Statistical Analysis

Statistical analyses and graph generation, including heatmaps, were performed using GraphPad Prism version 8.4.3 (GraphPad Software Inc., La Jolla, CA, USA). For mouse experiments, each individual mouse was considered an independent biological replicate. Comparisons among the three mouse treatment groups were performed using one-way ANOVA followed by Tukey’s multiple-comparisons test. Correlations between Foxp3-positive cell numbers and histological parameters were assessed using Pearson’s correlation coefficient. Data distributions were assessed using the Shapiro–Wilk test. For the Ki67 dataset, for which a significant deviation from normality was detected in one group, the Kruskal–Wallis test followed by Dunn’s multiple-comparisons test and Spearman’s rank correlation analysis were additionally performed as sensitivity analyses. For the biofilm formation assay, the mean value of the technical triplicates from each independent experiment was used for statistical analysis, and comparisons were performed using one-way repeated-measures ANOVA with the Geisser–Greenhouse correction, followed by Tukey’s multiple-comparisons test. A *p*-value of <0.05 was considered statistically significant. Detailed statistical results, including effect estimates, 95% confidence intervals, exact *p*-values, and multiplicity-adjusted *p*-values, are provided in Supplementary Tables S8 and S9.

## 3. Results

### 3.1. ASV Analysis of the Tumor-Associated Microbiota Identified Candidate Bacterial Species

We previously analyzed the microbiota profiles of oral cancer tumor tissues, saliva, dental plaque, and tongue coating from patients with OSCC and demonstrated that *Fusobacterium nucleatum* was relatively enriched in the tumor tissue samples [25]. We also reported the potential involvement of *F. nucleatum* subsp. *polymorphum* in OSCC progression [25]. However, in addition to *F. nucleatum*, a diverse range of oral commensal bacteria is present within OSCC tumor tissues. Therefore, to identify bacterial species potentially involved in shaping the tumor microenvironment, we reanalyzed our previously published 16S rRNA gene sequencing dataset at the amplicon sequence variant (ASV) level [25].

**Figure 1:**
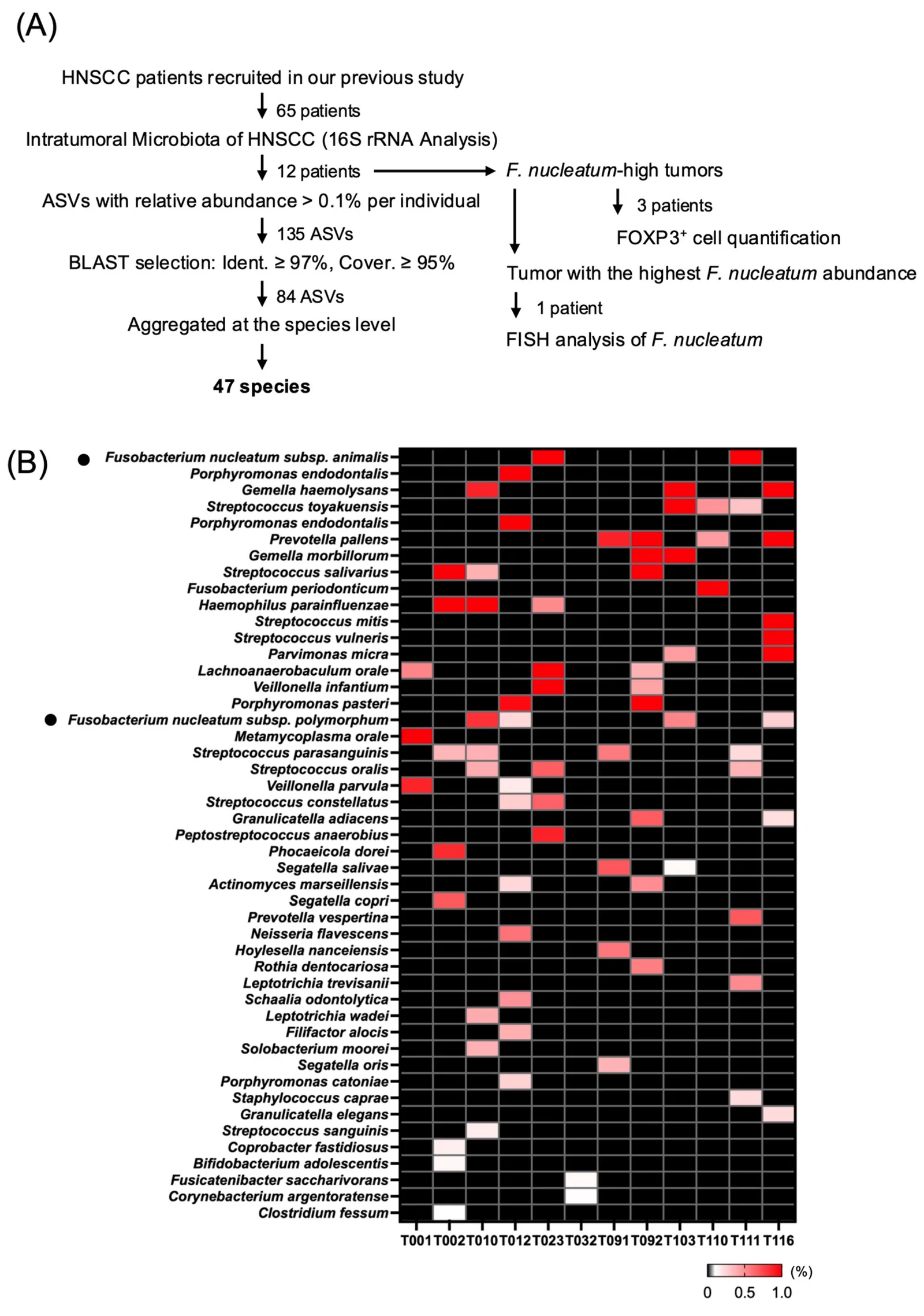
ASV-level reanalysis identifies candidate bacterial species in the tumor-associated microbiota of oral and pharyngeal squamous cell carcinoma. (**A**) Workflow of the ASV-level reanalysis of 16S rRNA gene sequencing data from the tumor-associated microbiota of patients with oral and pharyngeal squamous cell carcinoma (SCC). Among the 65 patients recruited in our previous study, tumor tissue samples were available from 12 patients. A total of 2302 amplicon sequence variants (ASVs) detected in tumor tissues from 12 patients were reanalyzed. ASVs with a relative abundance ≥ 0.1% in individual samples (135 ASVs) were subjected to BLASTn analysis. ASVs showing ≥ 97% sequence identity and ≥95% query coverage were assigned to the species level (84 ASVs). Relative abundances of ASVs assigned to the same species were aggregated, resulting in 47 candidate bacterial species for subsequent analyses. Among the 12 tumor samples, *F. nucleatum* was detected by 16S rRNA analysis in three samples, which were used for FOXP3^+^ cell quantification; the sample with the highest *F. nucleatum* abundance was further subjected to FISH analysis. (**B**) Heatmap showing the relative abundance (%) of the 47 candidate bacterial species identified in (**A**) across tumor tissues from the 12 tumor samples. Each column represents one patient and each row represents one bacterial species.

Among the 65 patients with oral and pharyngeal squamous cell carcinoma recruited in our previous study [25], sufficient residual tumor tissue for 16S rRNA gene analysis was available from 12 patients (Table 1). ASVs (amplicon sequence variants) comprising the tumor-associated microbiota were obtained from the 16S rRNA gene sequencing data of these 12 tumor tissues. Among a total of 2302 ASVs, 135 ASVs with a relative abundance of ≥0.1% in at least one tumor sample were selected for further analysis. These ASVs were subsequently subjected to BLASTn analysis, and 84 ASVs showing ≥ 97% sequence identity and ≥95% query coverage were retained for further taxonomic analysis. Finally, ASVs assigned to the same bacterial species were merged by summing their relative abundances, resulting in the identification of 47 bacterial species for subsequent analyses (Figure 1A).

Among the 47 bacterial species identified, ASVs most closely matching *F. nucleatum* subsp. *animalis* exhibited the highest relative abundance within the tumor tissues. Detailed BLASTn analysis of the eight Fusobacterium-associated ASVs identified two ASVs most closely matching *F. nucleatum* subsp. *animalis*, with 100% query coverage and 98.85% and 99.42% sequence identity to the corresponding reference sequence, respectively (Supplementary Figure S2). Because these assignments were based on short 16S rRNA amplicon sequences, they were interpreted as closest-match rather than definitive subspecies-level assignments. Other highly abundant taxa included oral commensal bacteria belonging to the genera *Porphyromonas, Gemella, and Streptococcus*. In addition, *F. nucleatum* subsp. *polymorphum,* which we previously identified as a potential tumor-promoting subspecies in OSCC, was also detected in the intratumoral microbiota (Figure 1B) [25].

Taken together, these results demonstrate that diverse oral commensal bacteria are present within the analyzed tumor tissues. Among the identified bacterial species, ASVs most closely matching *F. nucleatum* subsp. *animalis* exhibited the highest relative abundance, while *F. nucleatum* subsp. *polymorphum*, which had previously been implicated in OSCC progression, was also detected. Accordingly, reference strains of *F. nucleatum* subsp. *animalis* and *F. nucleatum* subsp. *polymorphum* were selected as the principal candidates for subsequent functional analyses.

### 3.2. Regulatory T Cells Preferentially Accumulated in the Peritumoral Region of OSCC Tissues

**Figure 2:**
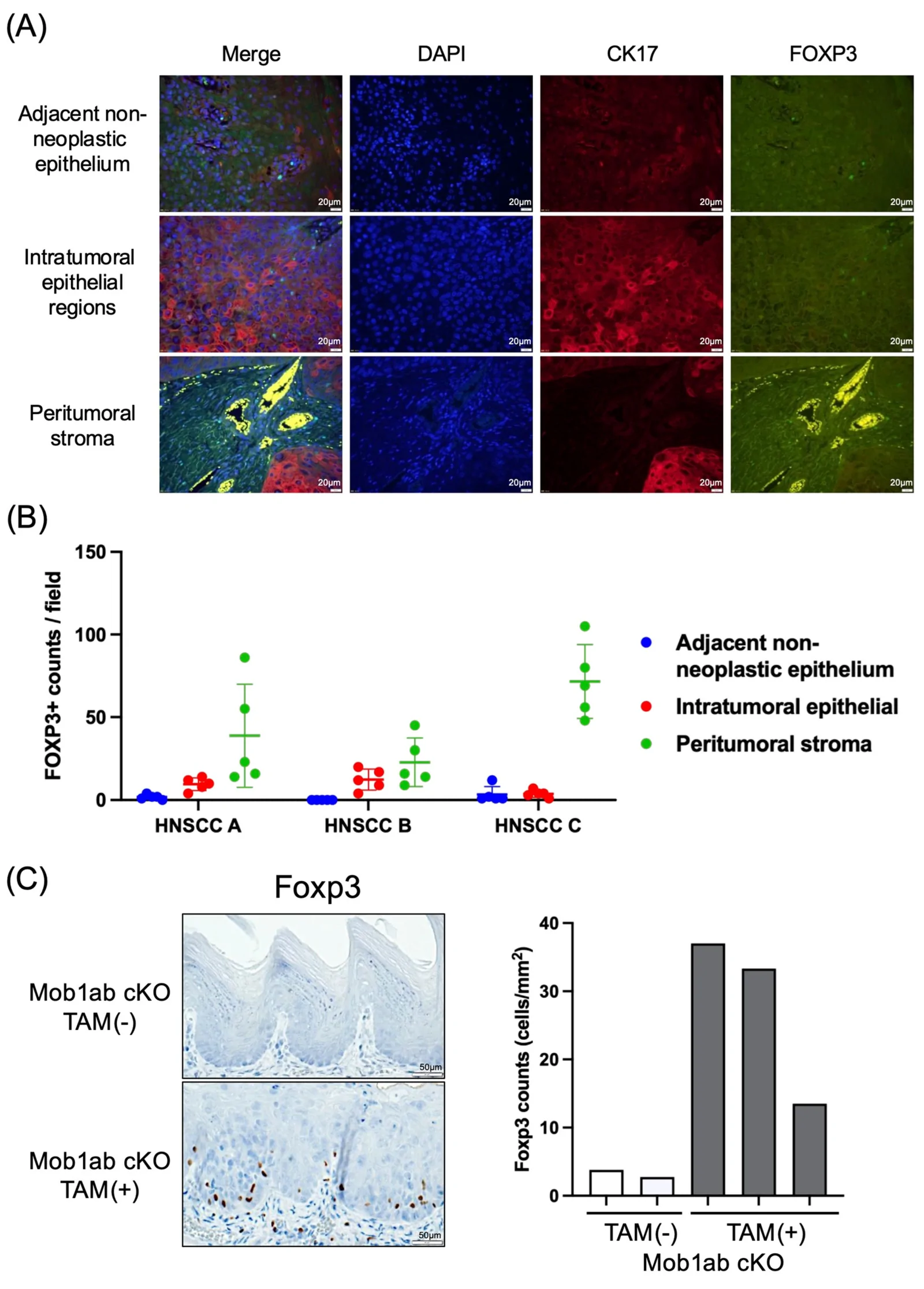
Distribution of FOXP3-positive cells in human OSCC and the Mob1a/b conditional knockout mouse model. (**A**) Representative immunofluorescence images of adjacent non-neoplastic epithelium, intratumoral epithelial regions, and peritumoral stroma in human OSCC tissue. Sections were stained for CK17 (red), FOXP3 (green), and DAPI (blue). Merged and individual fluorescence channels are shown. (**B**) Quantification of FOXP3-positive cells in the three histological compartments in three OSCC specimens in which *F. nucleatum* was detected by 16S rRNA analysis. Five fields were analyzed for each compartment in each specimen. Each dot represents one microscopic field, and horizontal lines indicate the mean ± SD of the five fields. (**C**) Representative immunohistochemical staining for Foxp3 in tongue tissues from tamoxifen-induced Mob1a/b conditional knockout mice. Representative images from two TAM(−) mice and three TAM(+) mice are shown. The bar graph shows the density of Foxp3-positive cells (cells/mm^2^) in individual mice.

Given the established role of Tregs in immunosuppressive tumor microenvironments and their regulation by bacterial metabolites [28,29], we examined the localization of regulatory T cells (Tregs) in OSCC patients’ tissues. Immunofluorescence staining was performed on human OSCC tissues using CK17 as a marker of neoplastic cancerous epithelial cells and FOXP3 as a marker of Tregs. Additional double immunofluorescence staining confirmed that FOXP3-positive cells in the peritumoral region co-expressed CD4, further supporting a Treg phenotype (Supplementary Figure S4B). To evaluate the spatial distribution of FOXP3-positive cells, three OSCC specimens in which *F. nucleatum* was detected by 16S rRNA analysis were analyzed. Representative images of adjacent non-neoplastic epithelium, intratumoral epithelial regions, and peritumoral stroma are shown in Figure 2A. FOXP3-positive cells were counted in five fields from each histological compartment in each specimen. In all three specimens, FOXP3-positive cells were more abundant in the peritumoral stroma than in the intratumoral epithelial regions or adjacent non-neoplastic epithelium (Figure 2B, Supplementary Figure S4A), supporting the preferential accumulation of FOXP3-positive cells in the peritumoral stromal compartment of human OSCC tissues.

Next, we examined the localization of Foxp3-positive cells in a mouse tongue carcinogenesis model. Similarly to the findings in human OSCC tissues, Foxp3-positive cells preferentially accumulated around the epithelial lesions (Figure 2C). Histologically, the lesions in tamoxifen-treated mice exhibited dysplastic changes but showed no evidence of invasive tumor growth, indicating that they represented preinvasive lesions rather than overt invasive carcinoma.

Taken together, these results suggest preferential accumulation of FOXP3-positive cells in the peritumoral stromal compartment of human OSCC tissues and increased Foxp3-positive cell accumulation in preinvasive tongue lesions in the mouse model. Also, these findings suggest that Treg accumulation may already occur in preinvasive lesions.

### 3.3. F. nucleatum Promoted Treg Differentiation and Produced Butyrate

**Figure 3:**
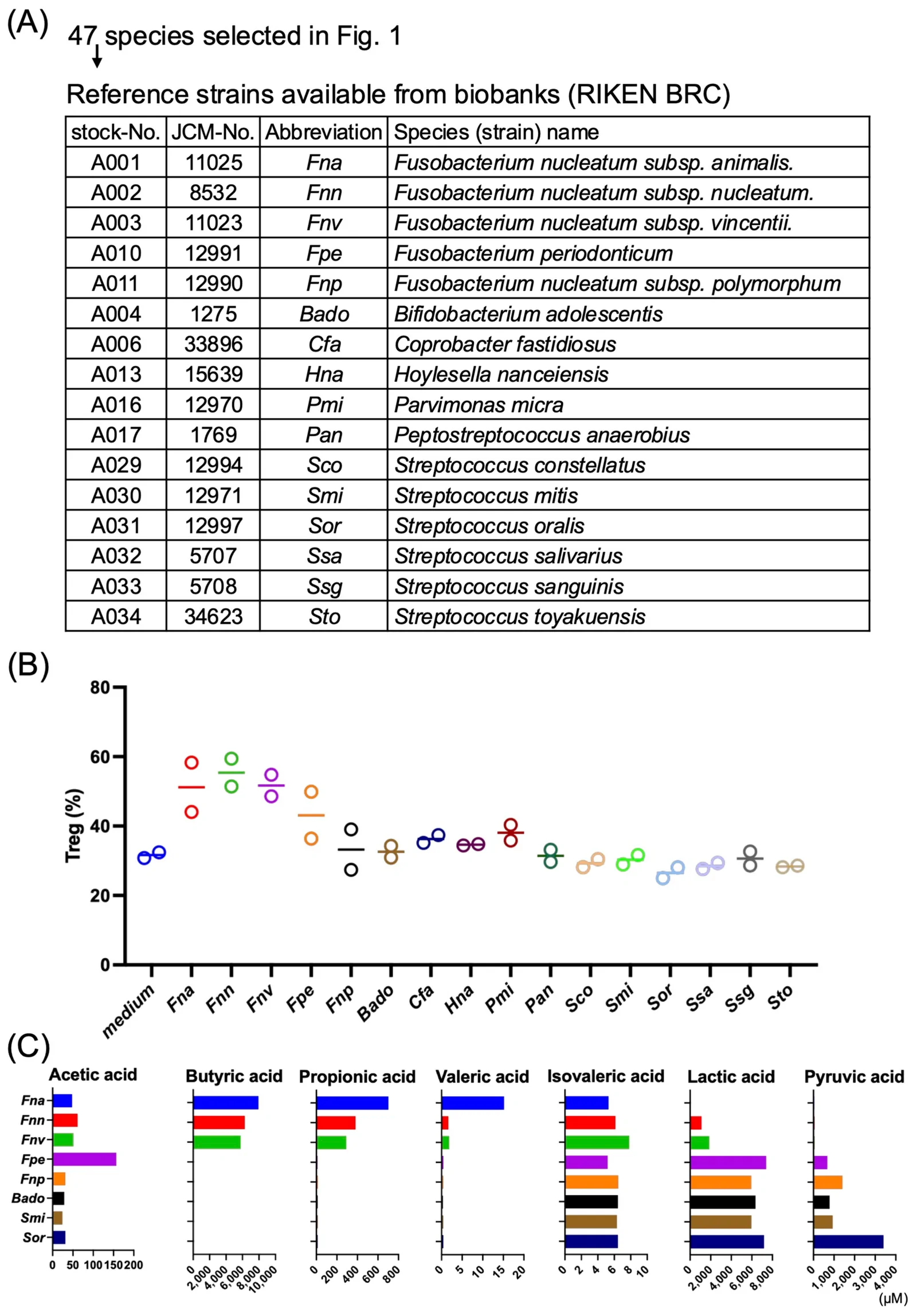
Selected bacterial strains induce regulatory T cells and produce short-chain fatty acids. (**A**) Selection of bacterial strains used for functional analyses. Among the 47 candidate bacterial species identified in Figure 1, 16 reference strains available from the RIKEN BioResource Research Center (JCM strains) were selected for subsequent in vitro experiments. (**B**) In vitro induction of regulatory T cells from naïve CD4^+^ T cells cultured in the presence of conditioned media from individual bacterial strains. The percentage of Foxp3^+^ cells among Zombie Dye^−^CD3ε^+^CD4^+^ cells is shown. The assay was independently performed twice using independently prepared T-cell samples and bacterial culture supernatants. Each point represents the mean of three technical replicate wells from one independent experiment, with two independent experimental values shown for each condition. The horizontal line indicates the mean of the two independent experimental values. (**C**) Concentrations of short-chain fatty acids and related organic acids in bacterial culture supernatants measured by LC-MS/MS. Metabolite concentrations are expressed as μM. The LC-MS/MS analysis was performed once, and the data are presented descriptively without inferential statistical analysis.

Because Tregs preferentially accumulated around OSCC lesions, we next investigated whether the candidate bacterial species identified in Section 3.1 influenced Treg differentiation in vitro. Among the 47 bacterial species identified in Section 3.1, those for which type strains were available from the RIKEN BioResource Research Center (RIKEN BRC, JCM) and could be cultured under identical anaerobic conditions were selected for functional analyses. Each bacterial strain was cultured anaerobically, and culture supernatants were collected after centrifugation. Naïve CD4-positive T cells were isolated from mouse spleens and lymph nodes and cultured under Treg-polarizing conditions in the presence of anti-CD3 antibody, anti-CD28 antibody, IL-2, and TGF-β. The culture supernatant from each bacterial strain was then added to the Treg differentiation system, and its effect on the generation of Foxp3-positive Tregs was evaluated (Figure 3A).

Culture supernatants derived from *F. nucleatum* exhibited subspecies-dependent effects on Treg differentiation. Specifically, culture supernatants from *F. nucleatum* subsp. *animalis*, *F. nucleatum* subsp. *nucleatum*, and *F. nucleatum* subsp. *vincentii* increased the frequency of Foxp3-positive Tregs compared with the control condition in both independent experiments (Figure 3B). In contrast, the culture supernatant from *F. nucleatum* subsp. *polymorphum* showed no apparent Treg-promoting activity. Likewise, culture supernatants from the other candidate bacterial species, including *Streptococcus* species, showed no apparent Treg-promoting activity.

Next, to investigate the relationship between Treg-promoting activity and bacterial metabolites, short-chain fatty acids and other organic acids in the bacterial culture supernatants were quantified by liquid chromatography–tandem mass spectrometry (LC-MS/MS). The analyzed metabolites included acetate, propionate, butyrate, valerate, isovalerate, lactate, and pyruvate. Among the analyzed metabolites, butyrate was detected in the culture supernatants of *F. nucleatum* subsp. *animalis*, *F. nucleatum* subsp. *nucleatum*, and *F. nucleatum* subsp. *vincentii*, all of which promoted Treg differentiation (Figure 3C). In contrast, little or no butyrate was detected in *F. nucleatum* subsp. *polymorphum*, which lacked Treg-promoting activity, or in the other candidate bacterial species. Lactate was detected at relatively high concentrations in several candidate bacterial species, including *Streptococcus* species. However, culture supernatants from these bacteria did not promote Treg differentiation. These findings suggest an association between butyrate production and Treg-promoting activity among the bacterial strains examined.

To further examine the Treg-promoting activity of butyrate, naïve CD4^+^ T cells were cultured in the presence of increasing concentrations of sodium butyrate. Butyrate increased Foxp3^+^ Treg differentiation in a concentration-dependent manner (Supplementary Figure S3B).

Taken together, these results show that the tested reference strains representing a subset of *F. nucleatum* subspecies exhibited both Treg-promoting activity and the ability to produce butyrate among the candidate bacterial species identified from the tumor-associated microbiota analyzed in this study. Notably, the reference strain of *F. nucleatum* subsp. *animalis*, corresponding to the subspecies most closely matching the ASVs with the highest relative abundance in the intratumoral microbiota, displayed both of these properties and was therefore selected for subsequent in vivo analyses.

### 3.4. Biofilm Formation by F. nucleatum Was Enhanced by Co-Culture with Streptococcus Species

To investigate whether interactions with resident oral bacteria enhance the biofilm-forming ability of *F. nucleatum,* we compared biofilm formation by *F. nucleatum* subsp. *animalis* and *F. nucleatum* subsp. *polymorphum* cultured alone or in co-culture with the oral resident bacteria *Streptococcus oralis* or *Streptococcus salivarius*. Biofilm formation was quantified by measuring the intensity of crystal violet staining (Supplementary Figure S1).

Both *F. nucleatum* subsp. *animalis* and *F. nucleatum* subsp. *polymorphum* formed biofilms when cultured alone. However, co-culture with *S. salivarius* significantly increased biofilm biomass for both *F. nucleatum* subspecies, whereas co-culture with *S. oralis* significantly increased biofilm biomass for *F. nucleatum* subsp. *polymorphum* but not for *F. nucleatum* subsp. *animalis* (Supplementary Figure S1). These results indicate that co-culture of *F. nucleatum* with resident oral *Streptococcus* species can enhance biofilm biomass, although the effect differed depending on the bacterial combination.

### 3.5. F. nucleatum subsp. animalis Promoted Treg Accumulation in a Mouse Tongue Carcinogenesis Model

**Figure 4:**
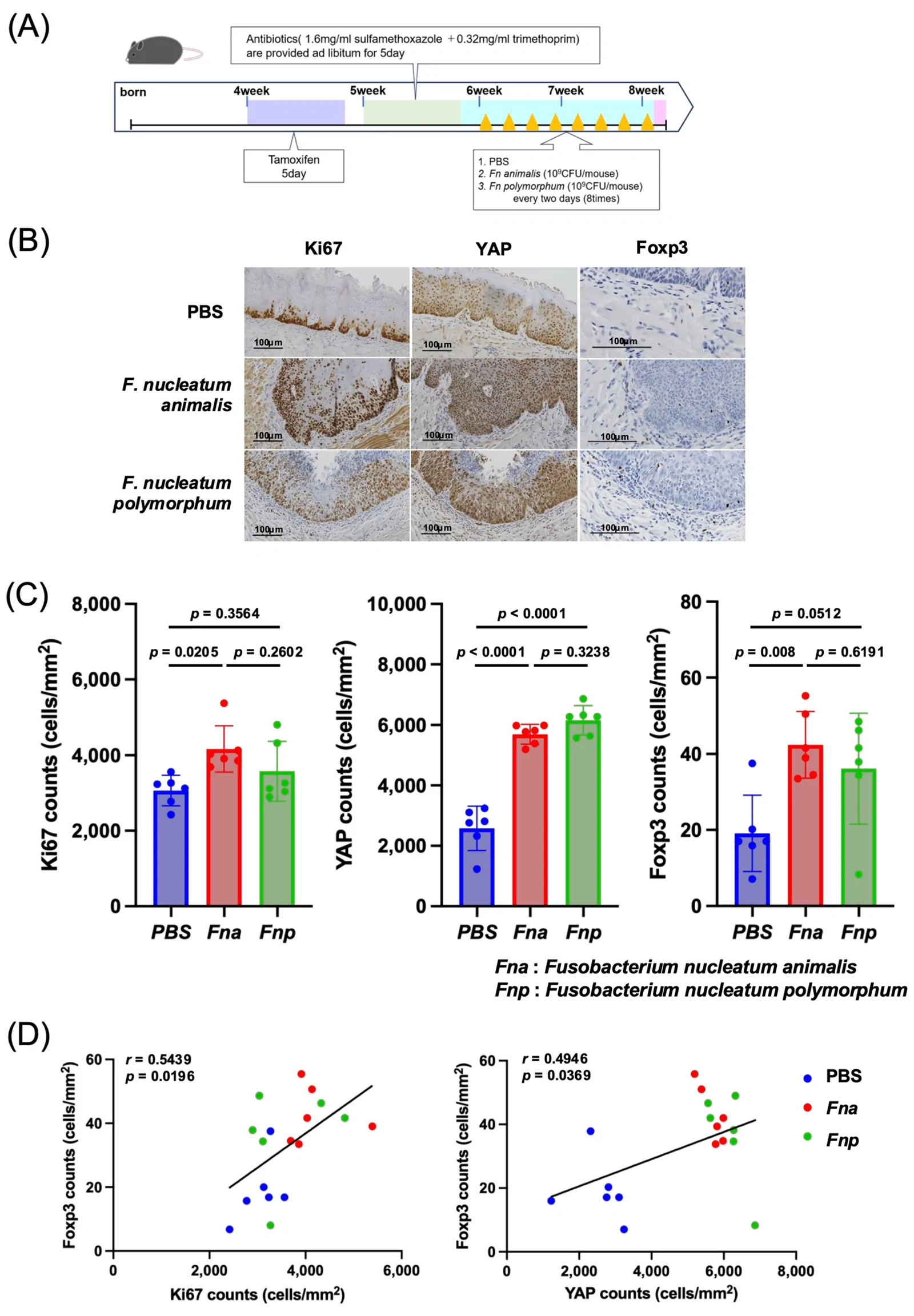
*Fusobacterium nucleatum* subspecies promote epithelial proliferation, YAP activation, and regulatory T-cell accumulation in the mouse tongue cancer model. (**A**) Experimental scheme. Tamoxifen-induced Mob1a/b conditional knockout mice were orally administered PBS (vehicle), *Fusobacterium nucleatum* subsp. *animalis* (Fna), or *F. nucleatum* subsp. *polymorphum* (Fnp). (**B**) Representative immunohistochemical images of Ki67, YAP, and Foxp3 staining in tongue tissues from each experimental group. (**C**) Quantification of Ki67-positive epithelial cells, nuclear YAP-positive cells, and Foxp3-positive cells. Data are presented as mean ± SD (*n* = 6 mice per group). Statistical analysis was performed using one-way ANOVA followed by Tukey’s multiple comparisons test. (**D**) Pearson correlation analysis of the quantified data shown in (**C**). Correlations between Foxp3-positive cell density and Ki67-positive epithelial cells, and between Foxp3-positive cell density and nuclear YAP-positive cells, were evaluated. Each dot represents an individual mouse. Pearson’s correlation coefficient (r) and two-tailed *p* values are shown.

To investigate whether *F. nucleatum* subsp. *animalis*, which promoted Treg differentiation in vitro, also induces Treg accumulation in vivo, we performed bacterial inoculation experiments using a mouse tongue carcinogenesis model [27]. *F. nucleatum* subsp. *polymorphum*, which has previously been suggested to promote OSCC progression [25], was analyzed in parallel for comparison (Figure 4A). The in vivo experiments were performed using *Fusobacterium* monoculture.

Mice in the tongue carcinogenesis model described in Section 3.2 received local inoculation of either *F. nucleatum* subsp. *animalis* or *F. nucleatum* subsp. *polymorphum*, whereas control mice received vehicle alone. Tongue tissues were subsequently collected, and the numbers of Ki67-positive and nuclear YAP1-positive cells were evaluated as indicators of epithelial proliferation and YAP pathway activation, respectively. Compared with the control group, administration of *F. nucleatum* subsp. *animalis* significantly increased both Ki67-positive and nuclear YAP1-positive cell numbers (Figure 4B,C). In contrast, administration of *F. nucleatum* subsp. *polymorphum* significantly increased the number of nuclear YAP1-positive cells, whereas the increase in Ki67-positive cell numbers did not reach statistical significance (Figure 4B,C).

Next, Foxp3-positive cells surrounding the lesions were evaluated. The number of Foxp3-positive cells was significantly increased in mice inoculated with *F. nucleatum* subsp. *animalis* compared with the control group (Figure 4C). In contrast, although the *F. nucleatum* subsp. *polymorphum* group showed a trend toward an increased number of Foxp3-positive cells compared with the control group, the difference did not reach statistical significance (Figure 4C). These results indicate differential effects of the two *F. nucleatum* subspecies on Treg accumulation.

To further examine the relationship between Treg accumulation and pathological changes, we analyzed the correlation between the number of Foxp3-positive cells and pathological parameters in individual lesions. The number of Foxp3-positive cells showed exploratory positive correlations with both the number of Ki67-positive cells and the number of nuclear YAP1-positive cells (Figure 4D). However, given the small sample size, these correlations should be interpreted cautiously and regarded as hypothesis-generating.

Collectively, these results demonstrate that *F. nucleatum* subsp. *animalis* significantly increased all three parameters examined, Ki67-positive, nuclear YAP1-positive, and Foxp3-positive cell numbers, indicating that it promoted YAP pathway activation and epithelial proliferation as well as Treg accumulation in the lesion microenvironment. In contrast, *F. nucleatum* subsp. *polymorphum* significantly increased the nuclear YAP1-positive area, but its effects on the Ki67-positive area and Foxp3-positive cell numbers were limited and did not reach statistical significance. Furthermore, the positive correlations between Foxp3-positive cell numbers and both the Ki67-positive area and nuclear YAP1-positive area may suggest an association between Treg accumulation and proliferative changes in the lesions, although these correlations should be interpreted as exploratory. These findings indicate that the effects of *F. nucleatum* on epithelial and immune parameters differ among subspecies, with *F. nucleatum* subsp. *animalis* showing more consistent effects on epithelial proliferation and Treg accumulation in this preinvasive tongue lesion model.

### 3.6. Spatial Association Between Treg Accumulation and Fusobacterium Nucleatum Localization in Human OSCC Tissues

Finally, we investigated the spatial relationship between Treg accumulation and *F. nucleatum* localization in human OSCC tissues. For this analysis, an FFPE tissue from the OSCC case with the highest relative abundance of *F. nucleatum* among the three *F. nucleatum*-positive specimens was selected. Serial tissue sections were prepared. One section was subjected to immunofluorescence staining using CK17 and FOXP3 to visualize tumor epithelial cells and Tregs, whereas the adjacent serial section was analyzed by fluorescence in situ hybridization (FISH) using the universal bacterial probe EUB338 together with the *F. nucleatum*-specific probe FUSO664 to visualize bacterial localization.

**Figure 5:**
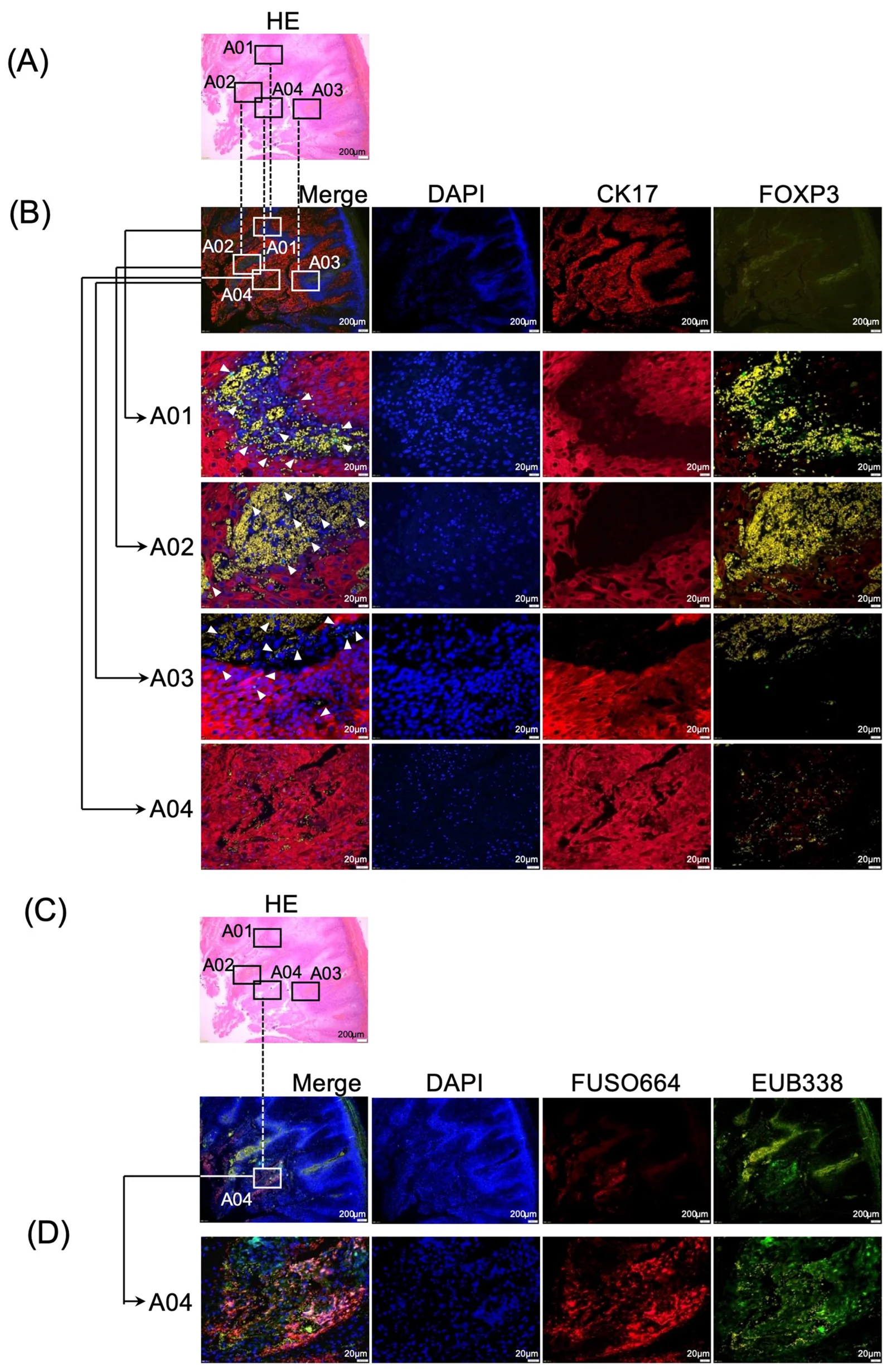
Spatial relationship between Fusobacterium nucleatum and FOXP3-positive cells in human OSCC tissues. (**A**) H&E-stained section showing the tumor tissue architecture and regions of interest (ROIs; A01–A04). (**B**) Immunofluorescence analysis of the corresponding tissue section stained for CK17 (red), FOXP3 (green), and DAPI (blue). The upper row shows a low-magnification overview with ROIs A01–A04 indicated. Higher-magnification images of A01–A04 are shown below as merged and individual fluorescence channels. Arrowheads indicate representative FOXP3-positive cells. (**C**) H&E-stained section indicating the regions corresponding to the FISH analysis. (**D**) FISH analysis of the adjacent serial section using the *F. nucleatum*-specific probe FUSO664 (red), the universal bacterial probe EUB338 (green), and DAPI (blue). The upper row shows a low-magnification overview, and the lower row shows a higher-magnification view of ROI A04 as merged and individual fluorescence channels.

The H&E-stained section was used to define regions of interest (A01–A04) within and around the tumor lesion (Figure 5A). Immunofluorescence analysis of the corresponding tissue section demonstrated heterogeneous spatial accumulation of FOXP3-positive cells around CK17-positive tumor regions, with prominent FOXP3-positive cell accumulation in selected peritumoral regions (Figure 5B). In the corresponding adjacent serial section, FISH analysis using EUB338 and FUSO664 detected bacterial signals in the tumor region (Figure 5C,D). Higher-magnification analysis of ROI A04 demonstrated FUSO664-positive signals overlapping with EUB338-positive bacterial signals within or immediately adjacent to the tumor region, confirming the presence of intratumoral *F. nucleatum* in this OSCC tissue specimen.

Taken together, these results demonstrate that, in this human OSCC case with high intratumoral *F. nucleatum* abundance, Treg accumulation and *F. nucleatum* localization were observed in spatial proximity within the tumor lesion (Figure 5). These findings support a spatial association between intratumoral *F. nucleatum* and Treg accumulation in this OSCC tissue specimen.

## 4. Discussion

In the present study, we reanalyzed the intratumoral microbiota of OSCC at the amplicon sequence variant (ASV) level and identified ASVs most closely matching *Fusobacterium nucleatum* subsp. *animalis* as the most abundant among the detected *F. nucleatum*-associated ASVs. We further found that FOXP3-positive cells preferentially accumulated around tumor lesions in human OSCC tissues and in a mouse tongue carcinogenesis model; that culture supernatants from selected *F. nucleatum* subspecies differed in their Treg-promoting activity in vitro and that this activity was associated with butyrate production; and that local administration of *F. nucleatum* subsp. *animalis* increased Foxp3-positive cell accumulation in the mouse model. In addition, spatial proximity between *F. nucleatum* and FOXP3-positive cells was observed in a selected human OSCC case with high intratumoral *F. nucleatum* abundance. The differential biological and immunological characteristics of *F. nucleatum* subsp. *animalis* and *F. nucleatum* subsp. *polymorphum* identified in this study are summarized in Table 2. Collectively, these findings support functional heterogeneity among *F. nucleatum* subspecies and suggest that selected *F. nucleatum* strains can influence epithelial and immune parameters relevant to the OSCC microenvironment under experimental conditions.

OSCC develops in a unique anatomical site that is continuously exposed to a complex oral microbiota. However, the bacterial composition within tumor tissues does not simply mirror that of the oral cavity. Our ASV-based analysis identified ASVs most closely matching *F. nucleatum* subsp. *animalis* as the most abundant among the detected *F. nucleatum*-associated ASVs, while other oral commensal bacteria, including *Streptococcus*, *Porphyromonas*, and *Gemella* species, were also detected. Many of these bacteria are recognized as members of the oral biofilm community. In particular, *F. nucleatum* functions as a central bridging organism that promotes coaggregation between early and late bacterial colonizers and thereby contributes to the structural organization of oral biofilms [30,31]. In addition, tumor tissues provide a distinct ecological niche characterized by hypoxia, acidic pH, tissue necrosis, nutrient gradients, and disruption of the epithelial barrier, conditions that may selectively favor colonization by anaerobic and tissue-invasive bacteria [4]. These observations raise the possibility that the OSCC microenvironment may influence the retention or enrichment of specific oral bacteria within tumor tissues. However, the present study cannot distinguish active bacterial colonization from preferential retention or accumulation within the tumor microenvironment.

An important aspect of the present study is that we examined functional differences among *F. nucleatum* subspecies rather than treating *F. nucleatum* as a single bacterial species. In the mouse tongue carcinogenesis model, *F. nucleatum* subsp. *animalis* significantly increased Ki67-positive and nuclear YAP1-positive cell numbers, whereas *F. nucleatum* subsp. *polymorphum* significantly increased nuclear YAP1-positive cell numbers but showed only a nonsignificant trend toward increased Ki67-positive cell numbers. However, repeated bacterial exposure itself may induce epithelial stress or injury and generalized inflammatory responses, which could secondarily influence epithelial proliferation, YAP activation, and immune-cell accumulation. Therefore, the present study cannot distinguish these nonspecific responses from direct tumor-specific effects of the administered *F. nucleatum* subspecies. In contrast, *F. nucleatum* subsp. *animalis* exhibited a greater capacity to induce Treg accumulation in vivo and Treg differentiation in vitro. Consistent with these findings, butyrate production was detected in the culture supernatants of the Treg-promoting *F. nucleatum* subspecies, whereas little or no butyrate was detected in *F. nucleatum* subsp. *polymorphum*. These observations suggest that the effects of *F. nucleatum* on epithelial parameters do not necessarily parallel its effects on the tumor immune microenvironment and that these biological activities may differ among *F. nucleatum* subspecies.

Previous genomic studies have demonstrated substantial heterogeneity among *F. nucleatum* subspecies and strains with respect to virulence factors, functional genes, and metabolic pathways [32]. More recently, specific clades of *F. nucleatum* subsp. *animalis* have been reported to be enriched in colorectal cancer tissues [13], supporting the concept that tumor-associated properties of *F. nucleatum* are determined at the subspecies or strain level. Taken together with these previous findings, our results further suggest that the biological effects of *F. nucleatum* are not mediated through a single mechanism but instead reflect functionally distinct properties among individual subspecies and strains.

These subspecies-specific differences may also have important clinical implications for understanding the role of *F. nucleatum* in OSCC. Previous studies have reported associations between the abundance of *F. nucleatum* in resected OSCC specimens and patient prognosis; however, these findings have not always supported a simple tumor-promoting role for this bacterium [14]. Most previous studies have evaluated the abundance of *F. nucleatum* in surgically resected tumors in relation to recurrence or survival, rather than addressing its potential contribution to the early stages of carcinogenesis or tumor progression. Moreover, analyses based solely on bacterial abundance cannot capture biologically important differences in *F. nucleatum* subspecies or strains, their spatial distribution within tumor tissues, interactions with coexisting microbiota, or their relationship with the local immune microenvironment.

Our findings suggest that understanding the biological significance of intratumoral *F. nucleatum* requires not only quantification of bacterial abundance but also characterization at the subspecies or strain level together with evaluation of their spatial localization and associated immune microenvironments. Such an integrated approach may provide a more comprehensive understanding of the role of intratumoral bacteria in OSCC biology.

With respect to Tregs, the present study demonstrated that Foxp3-positive cells preferentially accumulated in the peritumoral region rather than within the tumor nests in both human OSCC tissues and the mouse tongue carcinogenesis model. Although several studies have reported the prognostic significance of Tregs in OSCC, their biological and clinical significance appears to depend on their spatial localization and the status of the tumor immune microenvironment [19,20]. Our findings suggest that the peritumoral region represents an immunologically active niche where tumor cells, stromal cells, bacteria, and bacterial metabolites may interact to shape local immune responses.

Importantly, ASVs most closely matching *F. nucleatum* subsp. *animalis* were the most abundant among the detected *F. nucleatum*-associated ASVs, and the reference strain of *F. nucleatum* subsp. *animalis* exhibited Treg-promoting activity in vitro and increased Foxp3-positive cell accumulation in vivo. In the selected human OSCC case with high intratumoral *F. nucleatum* abundance, *F. nucleatum* was also observed in spatial proximity to FOXP3-positive cells. Together, these observations support the biological plausibility that *F. nucleatum*, including strains of subsp. *animalis*, may influence the local Treg-rich immune microenvironment.

In the present study, butyrate was identified as a bacterial metabolite associated with Treg-promoting *F. nucleatum* subspecies. Butyrate is well recognized as a microbial metabolite that promotes Treg differentiation in intestinal immunity [21,22,23] and our findings suggest that bacterially derived butyrate may similarly contribute to shaping the local immune microenvironment in OSCC. In addition, the biological effects of butyrate are highly context-dependent and vary according to the tissue microenvironment and cellular state. Within the tumor microenvironment, butyrate has been implicated not only in immune regulation but also in tumor cell proliferation, cellular senescence, the senescence-associated secretory phenotype (SASP), and metabolic adaptation. Indeed, butyrate produced by oral bacteria has been reported to induce cellular senescence, and the accompanying SASP may contribute to the establishment of an inflammatory and tumor-promoting microenvironment [33]. Therefore, the biological effects of *F. nucleatum* subsp. *animalis* may extend beyond Treg induction alone, and bacterially derived butyrate may influence tumor epithelial cells, stromal cells, and the broader tumor microenvironment.

In contrast to butyrate, lactate production was observed in several candidate bacteria but did not clearly correlate with their ability to promote Treg differentiation from naïve CD4-positive T cells under the present experimental conditions. These findings suggest that, at least in our experimental system, butyrate is more closely associated with the induction of Treg differentiation than lactate. Nevertheless, lactate remains an important metabolite in the tumor microenvironment. Previous studies have shown that lactate contributes to Treg maintenance and activation, metabolic adaptation under glucose-deprived conditions, and the expression of immune checkpoint molecules [34,35]. Moreover, lactate produced by cancer cells accumulates as a consequence of the Warburg effect and has been implicated in angiogenesis, immunosuppression, bacterial metabolism as a metabolic substrate, and biofilm formation. These observations suggest that, although lactate was not clearly associated with Treg differentiation in our experimental system, it may contribute to the maintenance or modulation of the immunosuppressive tumor microenvironment through mechanisms distinct from the initial induction of Treg differentiation.

Because *F. nucleatum* subsp. *animalis* significantly increased both Ki67-positive and nuclear YAP1-positive cell numbers, whereas *F. nucleatum* subsp. *polymorphum* significantly increased nuclear YAP1-positive cell numbers but not Ki67-positive cell numbers, epithelial effects independent of Treg induction should also be considered. Indeed, although *F. nucleatum* subsp. *animalis* showed more consistent effects on both epithelial and immune parameters, the differential effects observed between the two subspecies suggest that epithelial responses and immune modulation may represent distinct biological properties of *F. nucleatum* subspecies.

*F. nucleatum* possesses several well-characterized virulence factors, including FadA and Fap2, which have been implicated in tumor cell adhesion, activation of intracellular signaling pathways, and suppression of antitumor immune responses [9,10,11]. In addition, activation of the YAP pathway has been associated with *F. nucleatum*-mediated epithelial proliferation [25,27]. Therefore, the biological effects of *F. nucleatum* may involve multiple mechanisms, including, but not limited to, butyrate-associated immune modulation, direct interactions with tumor cells and immune cells through bacterial virulence factors, activation of YAP signaling, and interactions with coexisting members of the intratumoral microbiota.

The present study has several limitations. First, although butyrate production was closely associated with the Treg-promoting *F. nucleatum* subspecies, we did not directly demonstrate that butyrate itself mediates Treg differentiation. Because bacterial culture supernatants contain numerous bioactive components, including metabolites other than butyrate, extracellular vesicles, peptides, and nucleic acids, the contribution of these factors cannot be excluded. In addition, pH-matched controls and quantitative assessment of total cell recovery were not included in the conditioned-supernatant experiments; therefore, potential effects of differences in pH or overall cell recovery on the observed Treg differentiation cannot be completely excluded. Future studies should directly evaluate the role of butyrate by examining GPR43/GPR109A signaling, histone deacetylase (HDAC) inhibition, and epigenetic regulation of the *Foxp3* locus, all of which have been implicated in Treg differentiation and activation [21,22,23]. Second, although the present study identified functional differences among selected *F. nucleatum* subspecies, the in vivo analyses focused primarily on reference strains of *F. nucleatum* subsp. *animalis* and *F. nucleatum* subsp. *polymorphum*. Future studies using patient-derived isolates, comparative genomic analyses, and additional subspecies and strains will be necessary to clarify the bacterial determinants responsible for their distinct biological properties. Third, subspecies-level assignment based on short 16S rRNA amplicon sequences has inherent limitations in distinguishing closely related *F. nucleatum* subspecies, and higher-resolution approaches will be required for definitive taxonomic validation. However, independent validation by subspecies-specific qPCR or higher-resolution sequencing could not be performed because of the limited availability of residual biopsy DNA. Although FISH analysis confirmed the presence of *F. nucleatum* in the selected human OSCC tissue specimen, the probes used in this study could not discriminate individual *F. nucleatum* subspecies. Because the FISH analysis was performed in a single selected case with high intratumoral *F. nucleatum* abundance, this finding should be regarded as illustrative and cannot be generalized to OSCC as a whole. In addition, because this study retrospectively analyzed specimens from a previously established cohort, standardized histological-grade information and detailed oral-health variables were not available for all patients, limiting the clinicopathological characterization of the analyzed cohort. The development of subspecies-specific probes or complementary spatial metagenomic approaches will therefore be important to determine whether *F. nucleatum* subsp. *animalis* preferentially localizes to Treg-rich regions in human tumors. Finally, although epithelial proliferation, YAP activation, and Treg accumulation were evaluated in the mouse tongue carcinogenesis model, invasive tumor progression, metastasis, and long-term tumor outcomes were not examined. In addition, persistent bacterial colonization was not assessed, and the present study cannot exclude the possibility that features of the tumor microenvironment, such as hypoxia, tissue damage, or inflammation, may favor the retention of *F. nucleatum*, rather than bacterial accumulation being solely a cause of microenvironmental changes. Conversely, a Treg-rich immunosuppressive microenvironment may also facilitate bacterial persistence. Furthermore, CD4/FOXP3 co-staining supported the presence of a Treg phenotype in human OSCC tissues. However, as additional Treg-associated markers such as CD25, CTLA-4, and Helios and functional suppression assays were not included in the present study, the functional properties of these FOXP3-positive/CD4^+^FOXP3^+^ cells remain to be further characterized. Moreover, the functional contribution of Treg induction to tumor promotion remains to be directly established. Future studies combining Treg depletion strategies, inhibition of metabolite-related pathways, and long-term tumor progression models will further clarify the causal relationships between intratumoral *F. nucleatum*, immune modulation, and OSCC progression.

Regulatory T cells are essential for maintaining mucosal immune homeostasis by limiting excessive inflammatory responses against commensal microorganisms. However, once epithelial transformation has occurred, the same immunoregulatory mechanisms may inadvertently facilitate immune evasion by suppressing antitumor immunity. Our findings therefore suggest that *Fusobacterium nucleatum* subsp. *animalis* may contribute to the establishment of a Treg-rich immune microenvironment in preinvasive tongue lesions.

In addition to *F. nucleatum*, *Streptococcus* species were also identified within the intratumoral microbiota of OSCC, although they did not promote Treg differentiation under our experimental conditions. As early colonizers of the oral cavity, *Streptococcus* species initiate oral biofilm formation, whereas *F. nucleatum* functions as a bridging organism that promotes biofilm maturation (Supplementary Figure S1). Together with our supplementary findings showing increased biofilm biomass in selected combinations of *F. nucleatum* and *Streptococcus* species in vitro, these observations raise the possibility that cooperative bacterial interactions may influence biofilm formation and bacterial persistence. The biological significance of such polymicrobial interactions within tumor tissues, however, remains to be determined.

## 5. Conclusions

In conclusion, the present study identified ASVs most closely matching *F. nucleatum* subsp. *animalis* as the most abundant among the detected *F. nucleatum*-associated ASVs in OSCC and demonstrated that a reference strain of this subspecies exhibited Treg-promoting activity in vitro and increased Foxp3-positive cell accumulation in vivo. Together, these findings support the biological plausibility that selected intratumoral bacteria may influence the local immune microenvironment through bacterial metabolites and interactions with host cells and coexisting microbial communities. Future studies integrating subspecies- and strain-level microbiome analyses, spatial localization of intratumoral bacteria, bacterial metabolomics, and comprehensive immune profiling will be important for clarifying the role of intratumoral bacteria in OSCC biology and their potential clinical relevance.

## Figures and Tables

**Table 2:** Differential biological and immunological characteristics of *Fusobacterium nucleatum* subsp. *animalis* and *Fusobacterium nucleatum* subsp. *polymorphum* identified in this study.

Feature	*F. nucleatum* subsp. *animalis*	*F. nucleatum* subsp. *polymorphum*	Evidence
Relative abundance in human OSCC	Highest	Lower	Figure 1
Treg differentiation	Increased	No significant increase	Figure 3B
Butyrate production	Increased	No significant increase	Figure 3C
Biofilm formation (monoculture)	Detected	Detected	Supplementary Figure S1
Biofilm biomass in co-culture with *Streptococcus* spp.	Increased	Increased	Supplementary Figure S1
Ki67-positive epithelial cells	Significantly increased	No significant increase	Figure 4B,C
Nuclear YAP	Significantly increased	Significantly increased	Figure 4B,C
Foxp3-positive cells	Significantly increased	Trend toward an increase (*p* = 0.0512)	Figure 4C

**Note:** Descriptive terms are used instead of arrows to distinguish statistically significant increases from nonsignificant findings. Biofilm measurements reflect total crystal violet-stained biomass.

## Data Availability

The data presented in this study are openly available in the DDBJ Sequence Read Archive under BioProject accession number PRJDB16847.

## Supplementary Materials

The following supporting information can be downloaded at: https://www.mdpi.com/article/10.3390/microorganisms14092067/s1, Figure S1: Biofilm formation by *Fusobacterium nucleatum* subspecies in monoculture and dual-species culture with *Streptococcus* spp.; Figure S2: BLASTn-based taxonomic assignment of Fusobacterium-associated ASVs detected in tumor tissues; Figure S3: Flow cytometric gating strategy and concentration-dependent induction of Treg differentiation by butyrate; Figure S4: Spatial distribution and phenotypic characterization of FOXP3-positive cells in human OSCC tissues; Table S1: Source data for the heatmap shown in Figure 1B; Table S2: Source data for Figure 2B; Table S3: Source data for Figure 2C; Table S4: Source data for Figure 3B; Table S5: Source data for Figure 3C; Table S6: Source data for Figure 4C; Table S7: Source data for Supplementary Figure S1; Table S8: Detailed statistical analyses of the quantitative data presented in Figure 4; Table S9: Detailed statistical analyses of the biofilm data presented in Supplementary Figure S1.
