# Supplementary material for: Functional Heterogeneity of Fusobacterium nucleatum Subspecies in the Immune Microenvironment of Oral Squamous Cell Carcinoma
Source: Microorganisms. 2026 Sep 16;14(9):2067. doi: 10.3390/microorganisms14092067 (PMC13609895; doi:10.3390/microorganisms14092067)
Supplement: Supplementary file 1 [file microorganisms-14-02067-s001.zip › Supplementary Figures.pdf]

Supplementary Figures

Supplementary Figure S1.

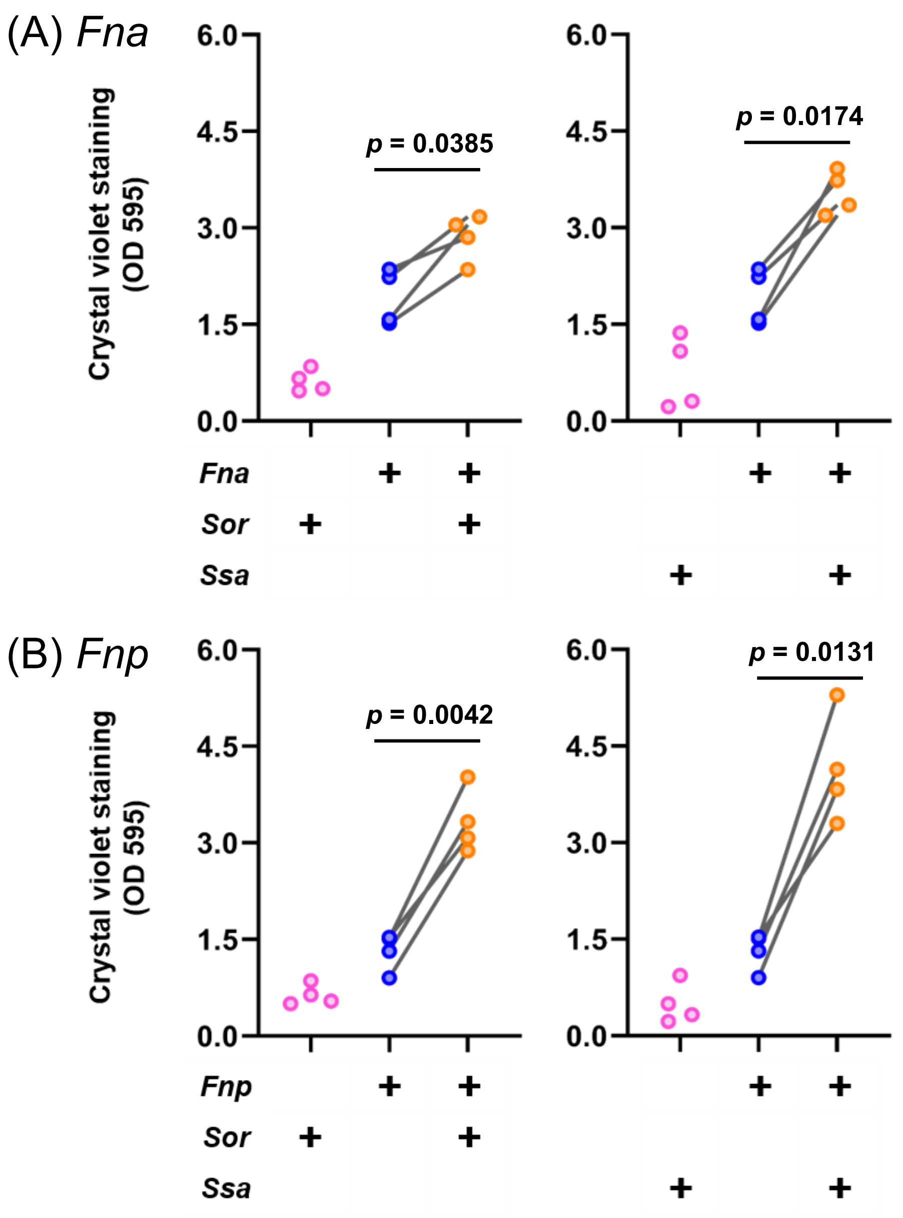

Supplementary Figure S1. Biofilm formation by *Fusobacterium nucleatum* subspecies in monoculture and dual-species culture with *Streptococcus* spp.

(A) Biofilm formation by *F. nucleatum* subsp. *animalis* (Fna) cultured alone or in dual-species culture with *Streptococcus oralis* (Sor; left) or *Streptococcus salivarius* (Ssa; right). (B) Biofilm formation by *F. nucleatum* subsp. *polymorphum* (Fnp) cultured alone or in dual-species culture with Sor (left) or Ssa (right). Biofilm biomass was quantified by crystal violet staining. Each point represents the mean OD_{595} value of three technical replicate wells from one of four independent experiments (n = 4). Lines connect measurements obtained in the same independent experiment. For each *F. nucleatum subspecies*, statistical comparisons were performed among the monoculture and the two corresponding dual-species cultures. Statistical significance was assessed using one-way repeated-measures ANOVA followed by Tukey's multiple-comparisons test. Samples were diluted fivefold before absorbance measurement, and OD_{595} values were corrected for the dilution factor.

Supplementary Figure S2.

The information of Fusobacterium's ASV in this study

Fn.No	ASVs ID	SILVA genus levels	Patient ID	ratio (%)	BLAST Top hit (species)	ASVs length (bp)
Fn_01	5c6fb905cf2c702e4baa007f63e616a6	*Fusobacterium*	T023	5.08	*Fusobacterium animalis*	347
Fn_02	ed85aa576d5a9baf64a367d2281912a6	*Fusobacterium*	T110	3.40	*Fusobacterium periodonticum*	334
Fn_03	5d454777c09b69d8c5f9be2eb4efac8c	*Fusobacterium*	T111	1.37	*Fusobacterium animalis*	347
Fn_04	8103e9cef935a53b595cca70b70d5fdb	*Fusobacterium*	T103	0.53	*Fusobacterium polymorphum*	334
Fn_05	4a284f0c19827db63a26e8d0fd76b657	*Fusobacterium*	T010	0.51	*Fusobacterium polymorphum*	334
Fn_06	e05e7b405e29d1b6a46b80999aa4a0e4	*Fusobacterium*	T010	0.31	*Fusobacterium polymorphum*	334
Fn_07	d45bf0ce878ee07e3e4ce905f3d6e567	*Fusobacterium*	T116	0.27	*Fusobacterium polymorphum*	334
Fn_08	622d6001e11b78f45d488a77f16b013d	*Fusobacterium*	T012	0.24	*Fusobacterium polymorphum*	334

Top5 best match species at BLASTn

Fn_01

Description	Max Score	Query Cover	E value	Per. Ident	Accession
Fusobacterium animalis strain JCM 11025 16S ribosomal RNA, partial sequence	619	100%	3.00E-177	98.85%	NR_113378.1
Fusobacterium animalis ATCC 51191 16S ribosomal RNA, partial sequence	599	94%	5.00E-171	99.69%	NR_117291.1
Fusobacterium nucleatum subsp. nucleatum ATCC 25586 16S ribosomal RNA, partial sequence	558	100%	8.00E-159	95.69%	NR_074412.1
Fusobacterium vincentii ATCC 49256 16S ribosomal RNA, partial sequence	558	100%	8.00E-159	95.68%	NR_117840.1
Fusobacterium simiae strain JCM 17465 16S ribosomal RNA, partial sequence	558	100%	8.00E-159	95.68%	NR_113318.1

Fn_02

Description	Max Score	Query Cover	E value	Per. Ident	Accession
Fusobacterium periodonticum strain ATCC 33693 16S ribosomal RNA, partial sequence	580	100%	2.00E-165	97.92%	NR_117839.1
Fusobacterium pseudoperiodonticum strain ChDC F213 16S ribosomal RNA, partial sequence	575	94%	7.00E-164	99.68%	NR_178363.1
Fusobacterium polymorphum ATCC 10953 16S ribosomal RNA, partial sequence	573	100%	3.00E-163	97.60%	NR_117842.1
Fusobacterium polymorphum ATCC 10953 strain JCM 12990 16S ribosomal RNA, partial sequence	573	100%	3.00E-163	97.60%	NR_113141.1
Fusobacterium hwasookii ChDC F128 16S ribosomal RNA, partial sequence	569	94%	3.00E-162	99.36%	NR_178362.1

Fn_03

Description	Max Score	Query Cover	E value	Per. Ident	Accession
Fusobacterium animalis strain JCM 11025 16S ribosomal RNA, partial sequence	630	100%	2.00E-180	99.42%	NR_113378.1
Fusobacterium animalis ATCC 51191 16S ribosomal RNA, partial sequence	599	94%	5.00E-171	99.69%	NR_117291.1
Fusobacterium nucleatum subsp. nucleatum ATCC 25586 16S ribosomal RNA, partial sequence	569	100%	4.00E-162	96.26%	NR_074412.1
Fusobacterium vincentii ATCC 49256 16S ribosomal RNA, partial sequence	569	100%	4.00E-162	96.25%	NR_117840.1
Fusobacterium simiae strain JCM 17465 16S ribosomal RNA, partial sequence	569	100%	4.00E-162	96.25%	NR_113318.1

Fn_04

Description	Max Score	Query Cover	E value	Per. Ident	Accession
Fusobacterium polymorphum ATCC 10953 16S ribosomal RNA, partial sequence	579	100%	6.00E-165	97.90%	NR_117842.1
Fusobacterium polymorphum ATCC 10953 strain JCM 12990 16S ribosomal RNA, partial sequence	579	100%	6.00E-165	97.90%	NR_113141.1
Fusobacterium polymorphum ATCC 10953 16S ribosomal RNA, partial sequence	558	100%	7.00E-159	96.41%	NR_041807.1
Fusobacterium periodonticum strain ATCC 33693 16S ribosomal RNA, partial sequence	553	100%	3.00E-157	96.44%	NR_117839.1
Fusobacterium gastrosuis strain CDW1 16S ribosomal RNA, complete sequence	551	100%	1.00E-156	96.44%	NR_146837.2

Fn_05

Description	Max Score	Query Cover	E value	Per. Ident	Accession
Fusobacterium polymorphum ATCC 10953 16S ribosomal RNA, partial sequence	601	100%	1.00E-171	99.10%	NR_117842.1
Fusobacterium polymorphum ATCC 10953 strain JCM 12990 16S ribosomal RNA, partial sequence	601	100%	1.00E-171	99.10%	NR_113141.1
Fusobacterium polymorphum ATCC 10953 16S ribosomal RNA, partial sequence	580	100%	2.00E-165	97.60%	NR_041807.1
Fusobacterium hwasookii ChDC F128 16S ribosomal RNA, partial sequence	569	94%	3.00E-162	99.36%	NR_178362.1
Fusobacterium polymorphum ATCC 10953 16S ribosomal RNA, partial sequence	569	94%	3.00E-162	99.36%	NR_117288.1

Fn_06

Description	Max Score	Query Cover	E value	Per. Ident	Accession
Fusobacterium polymorphum ATCC 10953 16S ribosomal RNA, partial sequence	595	100%	6.00E-170	98.80%	NR_117842.1
Fusobacterium polymorphum ATCC 10953 strain JCM 12990 16S ribosomal RNA, partial sequence	595	100%	6.00E-170	98.80%	NR_113141.1
Fusobacterium polymorphum ATCC 10953 16S ribosomal RNA, partial sequence	575	100%	7.00E-164	97.31%	NR_041807.1
Fusobacterium hwasookii ChDC F128 16S ribosomal RNA, partial sequence	569	94%	3.00E-162	99.36%	NR_178362.1
Fusobacterium polymorphum ATCC 10953 16S ribosomal RNA, partial sequence	569	94%	3.00E-162	99.36%	NR_117288.1

Fn_07

Description	Max Score	Query Cover	E value	Per. Ident	Accession
Fusobacterium polymorphum ATCC 10953 16S ribosomal RNA, partial sequence	601	100%	1.00E-171	99.10%	NR_117842.1
Fusobacterium polymorphum ATCC 10953 strain JCM 12990 16S ribosomal RNA, partial sequence	601	100%	1.00E-171	99.10%	NR_113141.1
Fusobacterium polymorphum ATCC 10953 16S ribosomal RNA, partial sequence	580	100%	2.00E-165	97.60%	NR_041807.1
Fusobacterium hwasookii ChDC F128 16S ribosomal RNA, partial sequence	569	94%	3.00E-162	99.36%	NR_178362.1
Fusobacterium polymorphum ATCC 10953 16S ribosomal RNA, partial sequence	569	94%	3.00E-162	99.36%	NR_117288.1

Fn_08

Description	Max Score	Query Cover	E value	Per. Ident	Accession
Fusobacterium polymorphum ATCC 10953 16S ribosomal RNA, partial sequence	584	100%	1.00E-166	98.20%	NR_117842.1
Fusobacterium polymorphum ATCC 10953 strain JCM 12990 16S ribosomal RNA, partial sequence	584	100%	1.00E-166	98.20%	NR_113141.1
Fusobacterium hwasookii ChDC F128 16S ribosomal RNA, partial sequence	569	94%	3.00E-162	99.36%	NR_178362.1
Fusobacterium polymorphum ATCC 10953 16S ribosomal RNA, partial sequence	564	100%	2.00E-160	96.71%	NR_041807.1
Fusobacterium periodonticum strain ATCC 33693 16S ribosomal RNA, partial sequence	558	100%	7.00E-159	96.74%	NR_117839.1

Figure S2. BLASTn-based taxonomic assignment of Fusobacterium-associated ASVs detected in tumor tissues. Eight ASVs classified as Fusobacterium at the genus level were subjected to BLASTn analysis. The upper table shows the ASV identifier, patient ID, relative abundance, top BLASTn hit, and ASV length. The lower tables show the top five BLASTn matches for each ASV, including maximum score, query coverage, E-value, percent identity, and accession number. Taxonomic assignments used in the present study were based on the top BLASTn hit.

Supplementary Figure S3.

(A)

(B)

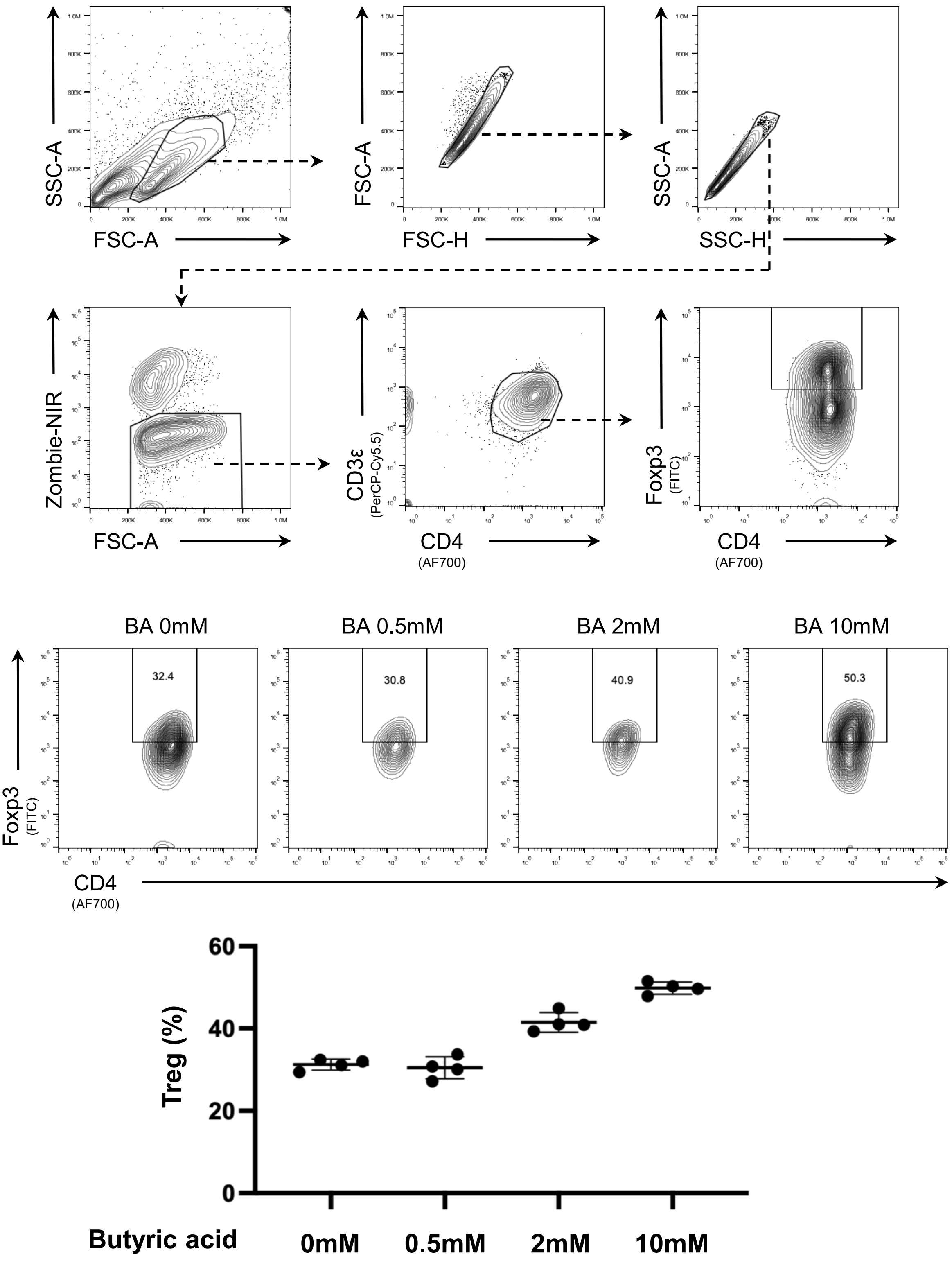

Figure S3. Flow cytometric gating strategy and concentration-dependent induction of Treg differentiation by butyrate.

(A) Representative flow cytometric gating strategy used to assess Treg differentiation. Cells were sequentially gated based on FSC/SSC characteristics, singlets, Zombie NIR-negative viable cells, and CD3ε$^+$CD4$^+$ T cells, followed by quantification of Foxp3$^+$ cells.

(B) Naïve CD4$^+$ T cells were cultured under Treg differentiation conditions in the presence of the indicated concentrations of sodium butyrate (0, 0.5, 2, and 10 mM) for 3 days. Representative flow cytometry plots and the percentage of Foxp3$^+$ cells among Zombie NIR$^-$CD3ε$^+$CD4$^+$ cells are shown. Data represent mean ± SD of quadruplicate wells from one representative experiment. The experiment was independently performed twice, with consistent results.

Supplementary Figure S4.

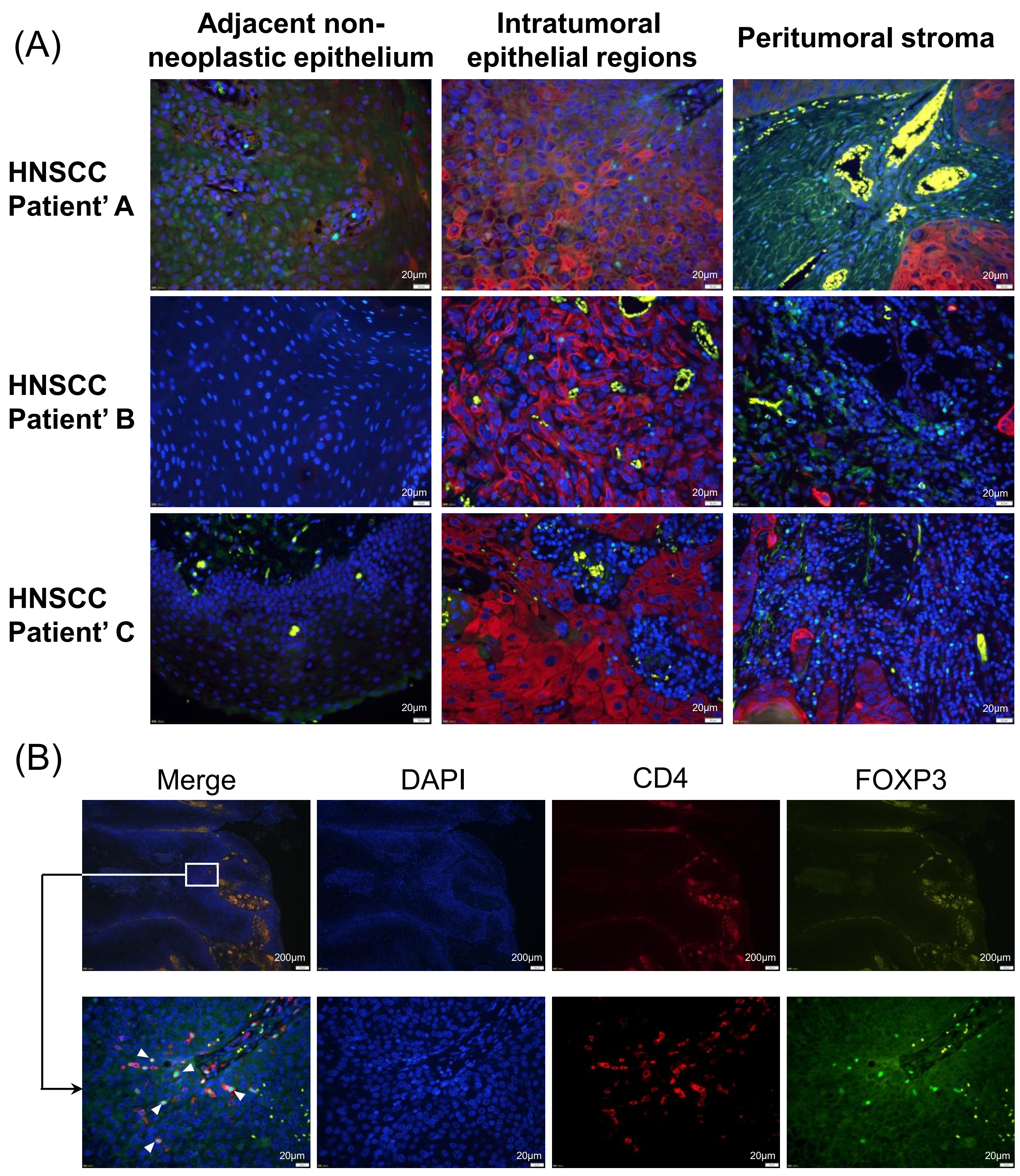

Supplementary Figure S4. Spatial distribution and phenotypic characterization of FOXP3-positive cells in human OSCC tissues.

(A) Representative merged immunofluorescence images of adjacent non-neoplastic epithelium, intratumoral epithelial regions, and peritumoral stroma from each of the three OSCC specimens analyzed in Figure 2B. Sections were stained for CK17 (red), FOXP3 (green), and DAPI (blue).

(B) Representative double immunofluorescence staining for CD4 (red) and FOXP3 (green) in human OSCC tissue. The upper row shows a low-magnification view, and the lower row shows a higher-magnification view of the indicated region. Arrowheads indicate representative $CD4^{+}FOXP3^{+}$ double-positive cells. DAPI (blue) was used for nuclear counterstaining.
